# Recent Advancements in the Technologies Detecting Food Spoiling Agents

**DOI:** 10.3390/jfb12040067

**Published:** 2021-11-27

**Authors:** Reena V. Saini, Prachi Vaid, Neeraj K. Saini, Samarjeet Singh Siwal, Vijai Kumar Gupta, Vijay Kumar Thakur, Adesh K. Saini

**Affiliations:** 1 Department of Biotechnology, MMEC, Maharishi Markandeshwar (Deemed to be University), Mullana, Ambala 133207, India; reenavohra10@mmumullana.org; 2 Department of Biotechnology, School of Sciences, AP Goyal Shimla University, Shimla 171009, India; prachi8427vaid@gmail.com; 3 School of Biotechnology, Jawaharlal Nehru University, New Delhi 110067, India; neeraj.einstein@gmail.com; 4 Department of Chemistry, MMEC, Maharishi Markandeshwar (Deemed to be University), Mullana, Ambala 133207, India; samarjeet6j1@gmail.com; 5 Biorefining and Advanced Materials Research Center, Scotland’s Rural College (SRUC), Kings Buildings, Edinburgh EH9 3JG, UK; Vijai.Gupta@sruc.ac.uk; 6 School of Engineering, University of Petroleum & Energy Studies (UPES), Dehradun 248007, India

**Keywords:** food-borne pathogens, aflatoxin, pesticides, aptasensor, biosensor, omics

## Abstract

To match the current life-style, there is a huge demand and market for the processed food whose manufacturing requires multiple steps. The mounting demand increases the pressure on the producers and the regulatory bodies to provide sensitive, facile, and cost-effective methods to safeguard consumers’ health. In the multistep process of food processing, there are several chances that the food-spoiling microbes or contaminants could enter the supply chain. In this contest, there is a dire necessity to comprehend, implement, and monitor the levels of contaminants by utilizing various available methods, such as single-cell droplet microfluidic system, DNA biosensor, nanobiosensor, smartphone-based biosensor, aptasensor, and DNA microarray-based methods. The current review focuses on the advancements in these methods for the detection of food-borne contaminants and pathogens.

## 1. Introduction

With the growing population, there is a dire need to address the food quantity, quality and safety issues [1,2,3]. Food is considered to be spoilt when any change occurs in the product quality, making it unacceptable for consumption by humans. Product quality and organoleptic properties of food material can be changed by a wide range of physical and chemical reactions. Some reactions and changes are also introduced because of specific enzymatic activity or due to the presence of microorganisms [4]. Apart from cross-contamination during the processes of harvesting or slaughter, specific properties of the food itself cause its spoilage, such as sensitivity to oxygen and light and the presence of various metabolites and endogenous enzymes. Primary changes in fresh food include oxidation of lipids and pigments, resulting in toxic and off-odor compounds, microbial contamination causing changes in pH, smell, and taste rendering the food unfit for consumption. The underlying mechanisms of spoilage are not well understood; therefore, many biochemical and chemical indices are set to estimate the spoilage and depreciated the food quality but still, the first analysis of spoilage is sensory assessment [5].

In one of its reports, FAO has stated that only two-thirds of the food produced is utilized for human consumption, and the other one-third is either spoiled or remains unused. This qualifies food spoilage as a global problem that needs to be addressed immediately [6]. Environmental and health issues further create more stress. For example, in the current scenario, it is estimated that the COVID-19 pandemic has created more stress on the population, and more people are undernourished than in the prior COVID-19 scenario. Additionally, healthy diets are costly and available to a limited population. More than 1.5 billion people are unable to get the basic level of essential nutrients [7]. Moreover, starting from producers to consumers, the spoilage of food results in food insecurity, leading to substantial economic losses to all the people involved in the chain.

Microbial contamination is one of the most common reasons for food spoilage. Ubiquitous and majorly microscopic microorganisms contaminate food products and remain unnoticed. High water-containing food gets spoilt by bacteria, while low water-containing food gets spoilt by molds and yeast. The shelf life of food is minimized by factors contributing more towards spoilage [8].

Spoilt food can be detected by employing multiple techniques, ranging from sensory detection to sensitive detection, for measuring even the low concentration of the contaminant. Many of the latest advancements have been made in food-contaminants detection, such as nanobiosensor, DNA biosensors, smartphone-based biosensors, aptasensor, DNA microarray, and single-cell droplet microfluidic systems. These techniques have been discussed in detail for the detection of biological and chemical contaminants.

## 2. Spoilage of Food by Adventitious Agents

### 2.1. Microorganisms

Foods with high protein and moisture content, such as milk, dairy products, poultry, fish, meat, and others, are nutritious, slightly acidic, or neutral in pH and, therefore, often become a breeding ground for the growth of microorganisms. The growth of these microorganism cause food spoilage, which gives the food industry a major economic loss, but, if these products reach the consumers, it causes significant health issues [4,9,10]. The microorganisms responsible for spoilage can be classified into broad categories, such as Gram-positive spore-forming bacteria, Gram-positive bacteria, Gram-negative rod-shaped bacteria, lactic acid bacteria, yeasts, and molds (Table 1). *Norovirus*, *Salmonella*, and *Escherichia coli* (*E. coli*) are the most common microorganisms responsible for different outbreaks and diseases. 

#### 2.1.1. Gram-Negative Rod-Shaped Bacteria

Due to gram-negative rod-shaped bacteria, food spoilage occurs mainly through the non-protein nitrogen (NPN) fraction of food product [11]. The NPN fraction of the food product is utilized first by the bacteria, followed by the metabolism of fatty acids and amino acids. Foods get spoilt owing to the release of certain enzymes resulting in the off-flavors and off-odors, the appearance of pigmented colonies, and slime. NPN can be efficiently utilized by *Pseudomonas*, *Aeromonas*, *Photobacterium*, *Shewanella*, and *Vibrio* [12]. *Vibrio* presents a unique feature where its halophilic nature causes the spoilage of cured meat and seafood [13].

It is also observed that the high moisture-containing food products (such as poultry, red meat, fish, milk, and dairy products) stored at natural pH under aerobic conditions are majorly affected by *Pseudomonas* spp. These pseudomonad strains belong to psychrotrophic organisms with a wide range of food sources that can be contaminated and utilized as substrate [11,14,15,16]. It forms a small proportion of fresh food initial microbial load (41%) and causes cystic fibrosis, respiratory and urinary infections, pneumonia as a hospital-acquired disease [17,18,19]. Spoilage at temperatures above 5 to 10 °C *Enterobacteriaceae* is more responsible as compared to *Pseudomonas.* Possible fecal contamination, inadequate processing, and post-processing contamination are the primary cause of Enterobacteriaceae members’ presence in spoilt food. Furthermore, due to its high pathogenicity, it causes multiple diseases, such as diarrhea, septicemia, bacteremia, respiratory disease, wound and burn infections, urinary tract infections, and meningitis [20].

#### 2.1.2. Gram-Positive Spore-Forming Bacteria

Microorganisms that survive chilling temperatures equivalent to 5 °C or less are capable of surviving the process of pasteurization or heating (*Bacillus* and *Clostridium* spp.) [21]. Gram-positive spore-forming bacteria grow slowly but are more resistant to high temperatures as compared to Gram-negative bacteria. *Clostridium* sp. That does not survive refrigeration temperatures (lower than 5 °C) are common contaminants in dairy products. At temperatures higher than 5 °C, they yield gas leading to the late blowing of the hard cheese during maturation [22,23]. However, apart from temperature, the pH and salt concentration of milk affects the bacterial spore germination, reproduction, and gas production capacity. In the case of *C. tyrobutyricum* strains, they do not produce gases at temperatures below 15 °C; hence, the spoilage does not occur below 15 °C [21,24]. 

*C. pasteurianum* strains spoilage was observed in different food products and was reported for the first time in figs, canned tomatoes, pears, pineapples, and peaches [25,26,27]. It is responsible for spoilage of acid foods because of its tolerance to the high amount of salt and sugar concentration even at acidic pH. It is also capable of spoiling shelf-stable apple juice [28]. These strains are heat resistant and, therefore, survive the heat treatment step of packaging. The optimum temperature for the growth of these bacteria is 35 °C, and maintaining the juice below pH 4.0 with mild heating during packaging can prevent the spoilage of apple juice [29,30,31]. 

Psychrotrophic and psychrophillic bacteria of clostridial species are responsible for spoilage of venison, lamb, and beef, rendering them inedible and causing financial losses to the producer. Psychro-clostridial species, such as *Clostridium algidixylanolyticum*, *C.algidicarnis*, *C. gasigenes*, *C. frigidicarnis*, and *C. estertheticum*, are known as the significant spoilers of red meat. These bacteria spoil the red meat during storage as they can grow at storage temperatures of −1.5 °C. Spoilt meat gets softened, produces foul odors, and also produces large amounts of drip exudates. Some bacteria also lead to gas production, as is the case for *C. estertheticum* and *C. gasigenes* [32]. Few Clostridial species were first found to be the causal organism for the spoilage of red meat in the fresh, chilled, vacuum-packaged, and sous-vide cooked form [33,34], having the main species *C*. *estertheticum* [35]. During processing, the spores of clostridial species enter vacuum packages where these spores germinate and lead to spoilage of food [36]. Therefore, it is also essential to understand that the quality of packaging material and the process shall also be monitored to mitigate the chances of contamination.

Bacteria belonging to *Bacillus* spp. are primarily aerobic and grow at 0 to 2 °C [32]. Spoilage of milk as bitty cream and sweet curdling may occur due to strains of *Bacillus* which grow at temperatures upto 5 °C, or even less [12,23,37]. Many *Bacillus* species are responsible for the spoilage of dairy products, but *B. cereus* is the only bacteria that causes food poisoning. *B. cereus* and *B. licheniformis* are the most prevalent species present in raw milk [38,39]. *B. licheniformis* causes spoilage of milk, affecting its organoleptic and functional properties, but does not qualify as a human pathogen [40,41]. It is capable of causing spoilage by the release of certain enzymes, but it does not produce biofilm, which is why it is found prevalently in the milk powders that are known to have a low spore count [42,43]. Another species, *B. sporothermodurans,* is found in Ultra High Temperature (UHT) processed milk and its products [44,45], and the enzymes released by it led to the spoilage of dairy products [46].

#### 2.1.3. Lactic Acid Bacteria

Lactic acid bacteria produce slime and CO_2_ as by-products in addition to producing lactic acid. Apart from the formation of foul odor, the flavors of food products, especially proteinaceous food items, such as vacuum-packed meat, poultry, and fish products, get spoilt [47]. They form a part of the initial microbial load but are not majorly responsible for their harmful impact on proteinaceous foods. These lactic acid-producing gram-positive, non-sporing rods bacteria are psychrotrophs cocci that include *Lactobacillus*, *Pediococcus*, *Leuconostoc*, *Streptococcus*, *Globicatella*, *Alloiococcus*, *Aerococcus*, *Dolosigranulum*, *Carnobacterium*, *Enterococcus*, *Lactococcus*, *Tetragenococcus*, *Oenococcus*, *Weissella*, and *Vagococcus* [48]. They act as a major spoiler in fermented food and beverages. Cloudiness in wine, fruit juices, and beer can be attributed to the lactic acid bacteria [49]. Some bacteria, such as *Lactobacilli*, are non-pathogenic, but, the others, such as *Lactobacillus lactis*, cause severe diarrhea, wounds, and urinary tract infection. *Leuconostoc* has been the cause of bacteremia, pneumonia, and cerebral hemorrhage in some clinical reports [50,51]. 

#### 2.1.4. Yeast and Molds

Yeasts and molds are organisms that can survive on multiple sources of nutrition, such as carbohydrates, pectin, organic acids, proteins, lipids, benzoate, propionate, and sorbate, and, therefore, are ubiquitous. They are resistant to extreme and unfavorable growth conditions, such as low pH, moisture, temperature, and in the presence of preservatives [52]. Contamination of yeast and mold has been reported [53] in packaged meats, fresh seafood, deli-type salads [54,55,56], and fresh vegetables [57]. The creation of slime and acids, pigmented growth on the surface of food products, and bad taste are major spoilage indicators due to infection by yeast and molds. Spoilage of food products by molds results in the release of mycotoxins by the mold that produces multiple toxic effects. [58]. Other fungal strains produce mycotoxins. Mycotoxin production in spoilt food is mainly due to the action of three fungal genera: *Aspergillus*, *Fusarium*, and *Penicillium* [59]. These mycotoxins cause oxidative stress-mediated DNA damage, ultimately decreasing cell viability [60]. Mycotoxins present in food products cause spoilage and major loss to the economy. The early detection of mycotoxins is crucial because of their infinitesimal concentration (parts per billion and nanograms) present in food products. They cause major clinical symptoms that often generate the need for high-cost treatments [61,62,63]. 

Spoilage of food due to physical or chemical methods is difficult to segregate because of similar characters of spoilage it produces, including oxidation and lipolysis. The chemical methods that cause spoilage have different levels to which it can spoil food products and different ranges of products that it can affect. 

### 2.2. Non-Biological Contaminants

Contamination in food can be caused due to contaminants other than biological contaminants. The contaminants introduced in food can either be natural or artificial or introduced during processing, packaging, transportation, and storage. Food-borne illness owing to these contaminants, such as cadmium, polychlorinated biphenyl (PCB), and lead, ranges from gastroenteritis to fatal disease and death cases [64].

Heavy metals deplete the essential nutrients present in the body in different forms and deter the host defenses. Contamination of heavy metal is known to cause malnutrition and an increased number of gastrointestinal diseases [65]. Industrial areas are more contaminated by heavy metals. Chemical food contaminants being toxic to a greater extent have been observed to act as carcinogens [66]. PCBs negatively affect neurological and immune system development in children [67]. Organic pollutants usually present in the waste produced by some industries and cause food spoilage are pesticides, such as chlordane, aldrin, DDT, by-products from the industry dibenzofurans and dibenzodioxins, and industrial chemicals, such as PCBs and HCBs [68,69]. The side effects of contamination by these species include an effect on reproductive systems, immune systems, and increased risk of diseases, such as cancer [70]. Besides organic pollutants, radioactive materials are also the cause of food spoilage [71]. They enter into soil, water bodies, and air and deplete their quality. The plants growing in the contaminated soil and the sea animals being utilized as food items often contain radionucleotides. Seafood contamination and drinking water contaminated with radioactive compounds have also been observed [72,73,74,75]. Regulatory bodies have decided the acceptable limits of these radionucleotides in different food products, and several studies are conducted by experimenting with specific experimental models to assess the safety in ingestion pathways [76].

Another source of contamination and spoilage of food is the food packaging material used for packaging and storing foods. The packaging types used are usually harmful plastics that are either previously contaminated with contaminants or the contaminants and toxins leach through the packaging. The leached products are known as migrants, and these contaminants led to specific, acute, or toxic effects. The risk posed by contaminants from plastic material is low, but the risk varies with temperature changes and contact time [77]. The shelf life of the product depends on the packaging method involved, and, among meat products, maximum shelf-life is in the case of modified atmosphere packaged meat [14]. The reason for increased shelf-life and decreased contamination by aerobic spoilage microorganisms is that the bags utilized for vacuum/ modified packaging are poorly permeable to oxygen and other gases.

## 3. Methods of Detection of Adventitious Agents in Food

The majority of food-borne illnesses are caused by *Salmonella*, *Cyclospora*, *Listeria monocytogenes*, *E. coli*, Hepatitis A, *Vibrio*, *Burkholderia cepacia*, and *Brucella* [78]. Food materials over a wide array can get contaminated by these adventitious agents, and proper understanding of them can help limit the contamination. One of the crucial factors preventing food spoilage is identifying the source/cause of the illness [47]. Many different detection methods have been utilized, and, with advancements made in technology and research, the detection methods are improved, as discussed below [79,80]. 

### 3.1. Single Cell Droplet Microfluidic System

Detection of a single cell in any bio-analytical process is crucial because every single droplet acts as an independent microreactor. Droplet microfluidics technology has the advantages of being high-throughput, parallelization, and integration. Droplet microfluidics have been utilized widely in microbial research and for the biological detection of biological entities, such as cancer biomarkers, exosomes, microbial extracellular products, and many more. Droplet-based microfluidic systems have been assessed for the cultivation and detection of microorganisms. Considering the utility of single-cell microfluidic technology, the detection of pathogens has been analyzed.

Microfluidic systems for detecting *Bacillus coagulans*, *Escherichia coli*, and *Listeria monocytogenes* have also been used to detect *Salmonella* [81,82,83,84]. Specific detection methods, such as enzyme-linked immunosorbent assay, colorimetric assays, nucleic acid-based assays, and SERS, are critical, time-consuming, costly, and tedious [85,86,87,88,89,90]. The latest sensitive detection methods include a droplet-based digital PCR method used for high sensitivity [81].

**Figure 1:**
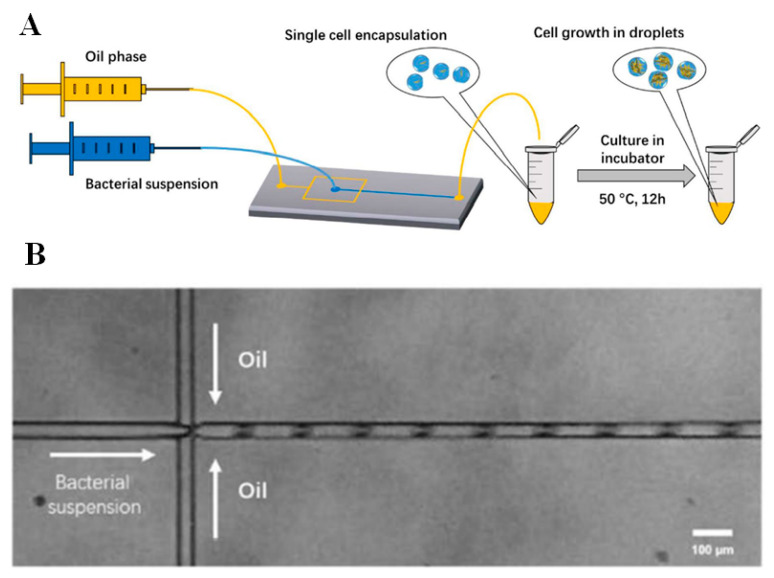
*B. coagulans* in microdroplet formation. (**A**) Of-chip cultivation of droplets of *B. coagulans*. (**B**) Bright-field image showing the microdroplets and flow of oil and bacterial suspension Scale: 100 μm. Adapted from Reference [81].

Detection of *B. coagulans* was performed by a microfluidic method where a flow-focusing microfluidic chip was used. Water in oil microdroplet was formed where cell suspension makes up the aqueous phase. Low polydispersity microdroplets were generated using the flow-focusing microfluidic device. The system gave 22% successful single-cell microdroplets. The growth pattern of the bacteria in the microfluidic system was also studied [81] (Figure 1).

Similarly, another study demonstrated a high throughput screening system that detects high lactic acid-producing bacteria *B. coagulans.* Cells were encapsulated in water-in-oil-in-water droplets followed by an analysis of high lactic acid producing microdroplets using a fluorescent reporter detecting the pH changes. The system also consists of sorting these high lactic acid-producing microdroplets by FACS analysis [82].

**Figure 2:**
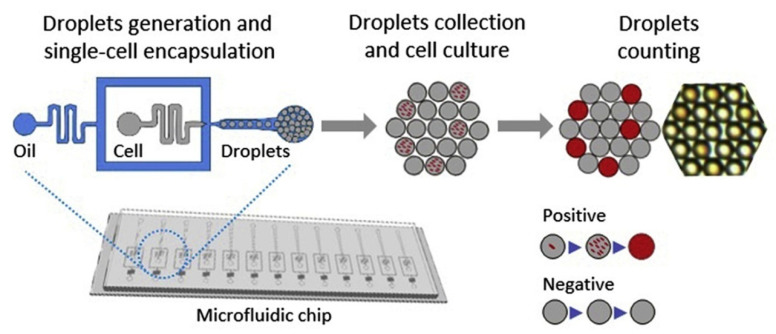
Single-cell droplet microfluidic system for the detection of *Salmonella.* The process has three steps: (a) Droplet generation and single-cell encapsulation of *Salmonella* through microfluidic system, (b) cell culture of collected droplets, and (c) analysis of fluorescent signal in the droplets. It is adapted from Reference [79] with permission from Elsevier (License Number 5184891131438), 2021.

In specific pathogens, such as Salmonella, the detection methods should be rapid, portable, and reliable, so, researchers explored a single-cell analysis microfluidic system. This protocol includes encapsulation of *Salmonella* into single-cell microdroplets containing growth medium with resazurin fluorescent dye, which aids in fluorescent detection of the pathogen within 5 h of microdroplet generation, collection, and incubation in culture. The detection limit of this system is 50 colony-forming units per ml within 5 h (Figure 2). The detection of the pathogen using this single-cell microfluidic method was performed in specific food samples, such as milk. For droplet generation, *Salmonella* was introduced with resazurin dye, milk sample, and growth medium. The detection time has been reduced from 24 h to 5 h. This method opens new avenues for researchers to increase the efficacy of detecting adventitious agents in food samples.

### 3.2. Analytical Devices-Based Onmicrofluidic Paper System

Microfluidic paper-based analytical devices (μPADs) were first explored in the year 2007 [91], and it is a boon for the developing nations because it provides a portable technology, with low risk and low-cost technology for disease screening in these areas. In contrast to the microfluidic analytical devices, which were designed using glass, silicon is designed in combination with super-polymer, μPADs which are designed using paper which reduces their cost [92,93,94,95,96,97,98,99,100]; thus, μPADs can be successfully applied in monitoring the disease condition, as well as in monitoring environmental contaminants [101,102]. These devices were also employed to measure the semi and/or quantitatively amount of an analyte by utilizing the standard, as well as sample, solutions. However, they cannot detect a meager amount of sample as it cannot be analyzed in the ppb or even ppt range [103].

### 3.3. Aptasensing for Detecting Microbes Their Toxins and Other Impurities

An emerging class of synthetic molecules includes single-stranded oligonucleotides usually synthesized using Systematic evolution of ligands by exponential enrichment (SELEX) and the class of molecules known as aptamer and can be utilized for the formation of biosensors with broad applicability [104,105,106,107]. Aptamers are more advantageous as compared to antibodies with properties, such as high thermal and chemical stability and low cost of production [108,109,110]. The sensors based on aptamers, called aptasensors, are often utilized for multiple applications, such as detecting certain toxins and contaminants in the food. *Aspergillus flavus* and *Aspergillus parasiticus* often produce toxins during their growth on food and feed, and such mycotoxins are named aflatoxins (AF) [111,112,113]. There are mainly six different types of aflatoxins [113,114]. 

With great technological advancements, many new techniques/assays are employed to detect and analyze aflatoxin. Aflatoxin, such as aflatoxin B1 (AFB1), has been detected by using techniques, such as high-performance liquid chromatography (HPLC), coupled with tandem mass spectroscopy (HPLC-MS/MS) [115], enzyme-linked immunosorbent assay (ELISA) [116], and HPLC, coupled with a fluorescence detector (HPLC-FLD) [117]. Development of an aptasensor for the detection of AFB1 utilizing RGO/MoS2/PANI@AuNPs-based electrochemical aptasensor exhibited advantageous properties, such as good stability, good selectivity, rapid response, and high sensitivity limits. It can be extended to determine the mycotoxins by controlling the functioning of the aptamer. The detection range for the aptasenso ris from 0.01 fg/mL to 1.0 fg/mL [118].

Aptamer recognition, coupled with molecular imprinting (MIP) recognition, was utilized as a double recognition method in aptasensor development [119]. The sensing interface involved in the aptamer is Au nanorod, which is helpful for its covalent immobilization with MIP. It was found that its recognition abilities were enhanced and were better than both aptamer recognition and MIP recognition alone. 

Selective detection of oxytetracycline, an antibiotic that can be a part of the food chain in edible products, was improved by synthesizing anew aptasensor, a sandwich-type electrochemical system. The aptasensor was based on a nanocomposite of graphene-three-dimensional nanostructure gold (GR3D-Au). In this sensor, the signal was amplified using nanoprobes of aptamer-AuNPs-horseradish peroxidase (HRP), and it improves the transfer of electrons and the loading capacity of the biomolecules. In coordination with the HRP modified gold nanoparticles, the aptamer leads to an excellent detection of oxytetracycline. The novel aptasensor has been applied to detect oxytetracycline in food samples, such as honey, and can be utilized for other food samples, as well [120].

In either organic or inorganic form, Arsenic is a typical heavy metal contaminant that acts as a toxin in multiple environmental sources, such as water, soil, various food stuff, vegetables, and cereals [121]. Amongst the different states of arsenic, As(III) is more toxic than As(V) or compounds of organic-As by a factor of 60 [122,123,124]. Health problems, such as skin damage, cardiac diseases, lung, and urinary bladder diseases, are witnessed in people consuming contaminated water [121,125,126,127]. This makes the detection of arsenic in water samples a very crucial element. 

Detection of As (III) has been improved by the introduction of various analytical methods, including HPLC [128], atomic fluorescence spectrometry [129], atomic absorption spectrometry [130,131], and electrochemical methods [132,133]. Electrochemical aptasensors have been used widely because of the advantageous properties that they offer. Another aptasensor was synthesized to detect arsenite As(III), which was based on3D reduced graphene oxide modified gold nanoparticles (3D-rGO/AuNPs). Additionally, a 5′-thiolate aptamer was synthesized and organized to detect As (III). It was assembled on a glassy-carbon-electrode, which is firstly modified with 3D-rGO/AuNPs, leading to the formation of Aptamer/3D-rGO/AuNPs/GCE. A covalent bond formation facilitates the modification between Au and S. If As (III) is present in any given sample, then, the ssDNA and the target interact to yield a G-quadruplex interaction, which produces a blockage for the transfer of electron. After the initial synthesis of these aptamers, the signals of the electrochemical impedance spectroscopy (EIS) were increased. Different parameters and conditions were optimized to advance the sensitivity of the aptasensor. This aptasensor was used for different water samples to detect As(III), specifically. The detection range of the aptasensor was 3.8 × 10^–7^–3.0 × 10^–4^ ng mL^−1^ [134]. Apart from detecting heavy metals and chemical contaminants in spoilt food, biological contaminants can also be detected using electrochemical aptasensors. 

Death due to medical sepsis is a significant problem, and the causative molecule is lipopolysaccharide (LPS). Electrochemical biosensor remains the method that can be used to identify the LPS best. Detection of lipopolysaccharide from *Escherichia coli* 055:B5 was enhanced by using an electrochemical aptasensor. The first step was the synthesis of rGO and gold nanocomposite (rGO-Au). Aptamer chains were then reacted with the rGO-Au nanocomposite and were immobilized on GCE. The modified electrode was characterized by using the voltammetry techniques, such as cyclic, square wave, and EIS. The designed electrochemical electrode was used to analyze serum of patients and healthy persons for the presence of LPS. It was found that it has higher sensitivity than the other designed electrochemical electrodes, and the specificity of the electrode is very high [107]. If Mg/CODs are used, that further increases the method’s sensitivity [107]. These sensitive techniques can be easily employed for raw materials and the detection of LPS in processed and packaged foods.

### 3.4. Electrochemical Biosensor Devices

Microbiological techniques used for conventional culturing of microorganisms are hectic, time-consuming, and slow. These techniques have been overpowered by high end-techniques, which involve the detection of food pathogens by incorporating biosensors that are fast, reliable, accurate, and specific. Biosensors enable real-time observation of a biological receptor compound (nucleic acid, enzyme, antibody, etc.) by incorporating a transducer. Biosensors are specific, leading to the detection of specific compounds from complex mixtures and complex food samples. Six major biosensors include mass biosensor, optical biosensor, magnetic biosensor, micromechanical biosensor, electrochemical biosensor, and thermal biosensor [135,136,137,138,139,140,141,142,143].

**Figure 3:**
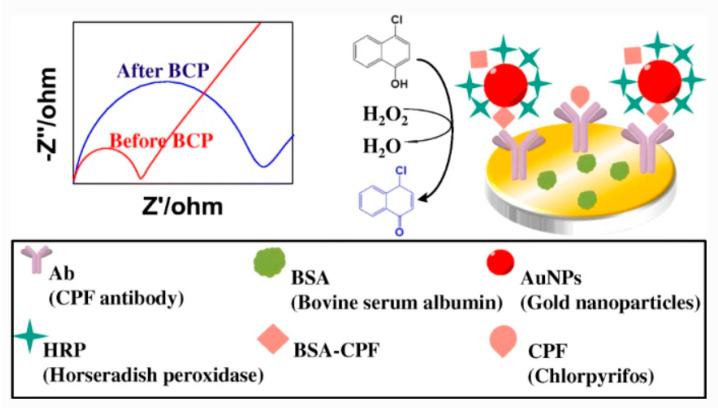
Chloropyrifos detection using competitive immunoassay method. Adapted from Reference [144] with prior permission from Springer Nature (License Number 5184890811012), 2021.

Chlorpyrifos is majorly available and the most crucial organophosphorus pesticide. The maximum residue limit has been defined in different food products for almost all the organophosphorus pesticides. If present in concentration more than the maximum residue limit, chlorpyrifos in food exhibits toxicity in humans. For detecting chlorpyrifos direct competitive-immunoassay can be used. In one study, gold nanoparticles (AuNPs) were used to fabricate the glassy carbon electrode (GCE), followed by binding with BSA and Antibody [144]. The chlorpyrifos was detected by the strategy of enzymatic biocatalytic precipitation amplification (BCP). Chlorpyrifos standards and the HRP-BSA complex were dropped onto the fabricated GCE at room temperature for half an hour. The formed electrode was then incubated in 1 mM 4-chloro-1-naphthol and 1 mM H_2_O_2_ mixture for 15 min. Impedimetric and cyclic voltammetry determination was performed. Cyclic voltammetry was determined within a voltage range of −0.20 and 0.60 V with a50 mV s^−1^ scan rate. The determination by the impedimetric method was performed at 0.22 V, alternating voltage of 10 mV and 10^−2^ to 10^6^ Hz as the frequency range (Figure 3) [1]. This method helped determine the pesticide in cabbage and lettuce and could be developed to determine other pesticides.

**Figure 4:**
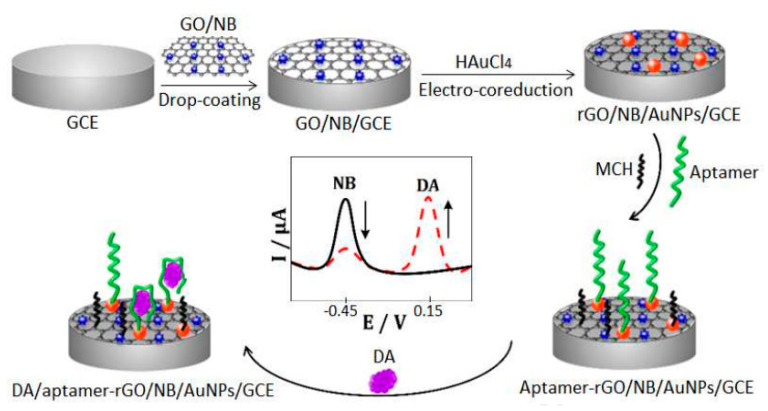
Fabricated aptamer MCH/aptamer-rGO/NB/AuNPs/GCE. Adapted from Reference [145] with permission from Elsevier (License number 5184900594504), 2021.

Electrochemical biosensors can be utilized for the detection of a wide array of molecules. One molecule that has been detected well using the electrochemical biosensor is dopamine. Graphene oxide and Nile blue were drop coated onto the glassy carbon electrode surface, forming GO/NB/GCE. AuNPs were electrodeposited onto the GO/NB/GCE by employing a one-step co-reduction treatment in conjunction with scanning using cyclic voltammetry. The electrodeposition of AuNPs caused the reduction of graphene oxide, resulting in rGO/NB/AuNPs/GCE formation. Along with this, the 5′-SH-terminated aptamer of dopamine was made to react with AuNPs in rGO/NB/AuNPs/GCE by the formation of bonds between Au and S, leading to the formation of aptamer-rGO/NB/AuNPs/GCE system. It was found that dopamine binds with the aptamer specifically; hence, the synthesized biosensor can be utilized to detect dopamine in patients (Figure 4). Apart from detecting specific clinical molecules, pesticides present in the food samples can also be analyzed [145]. In this case, the electrochemical cell was used for direct analysis of the pesticide. Carbaryl insecticide has been used extensively in agriculture for warding off an extensive range of insects. Carbaryl poisoning in humans can cause inhibition of cholinesterase, resulting in carbaryl poisoning [146]. For detecting these harmful components, the co-reduction of metal precursors was performed to synthesizeAu_x_Rh_1-x_ nanocrystals in the presence of oleylamine. The synthesized Au_42_Rh_58_ and, after characterizations, the Au_42_Rh_58_ nanocrystals modified electrode was made in two steps, by first making ink of Au_42_Rh_58_ reacting with carbon powder. The ink was then loaded as a thin film onto a glassy carbon electrode.

**Figure 5:**
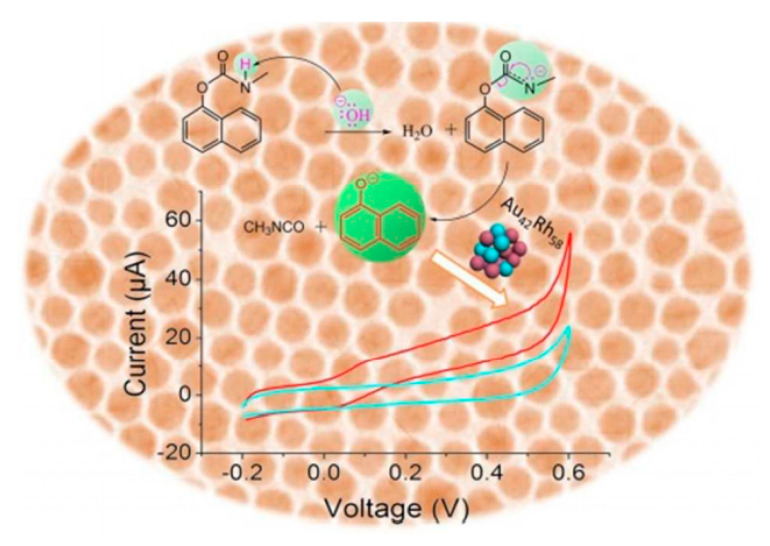
Detection of carbaryl using Au_x_Rh_x-1._ Reproduced from Reference [146] with permission from the Royal Society of Chemistry (Order no: 1160543), 2021.

During the detection of carbaryl, the C-O bond gets cleaved, which leads to the formation of its hydrolysis product 1-naphthol. The hydrolysis product thus formed undergoes electrocatalytic oxidation, which is then detected electrochemically. The specificity is very high, as can be seen with no interference from the presence of metal ions, organophosphate pesticides, glycine, serine, and aspartic acid. The electrocatalytic capabilities of Au_x_Rh_x−1_ of the bimetallic system were much higher than the monometallic systems, both Au and Rh. Out of the three different bimetallic compositions, Au_61_Rh_39,_ Au_42_Rh_58_, and Au_26_Rh_74_, Au_42_Rh_58_ has the best electrocatalytic capabilities [146] (Figure 5).

This bimetallic system of detection can be used to determine the extent of pesticides in various processed foods.

*Salmonella typhimurium* has been detected by an in-situ method [147]. It is a selective method, and it measures the oxygen mediated cathodic peak current during bacterial proliferation in cyclic voltammograms [147]. Bacterial pathogens can also be detected by the electrochemical biosensors, utilizing the presence of specific marker enzymes. The presence of coliform bacteria in water samples was analyzed by detecting the enzymes, such as β-D-glucuronide glucuronosohydrolase (GUS) and β-Dgalactosidase (β-GAL) [148,149].

### 3.5. Omics Tools for Detection: PCR-Based and LAMP-Based Detection

Omics encompasses areas of study, such as genomics, transcriptomics, proteomics, and metabolomics, which can be utilized for the rapid detection and take control measures of biological contaminants [150]. Omics has been applied for resolving the contamination by aflatoxins [151]. Omics tools analyze the biological contaminants present in any food sample by the analysis of the cellular RNA, DNA, proteins, and primary and secondary metabolites that are a part of the biological entity and facilitates the cellular pathways [150,152]. Array-based techniques have been introduced for the analysis of mycotoxin [150,153,154]. Initially, single mycotoxins were detected using the simple techniques of thin-layer chromatography (TLC); however, the detection of multiple mycotoxins was initiated using different techniques, such as HPLC, GC-MS, GC-MS/MS, LC-MS, LC-NMR-MS, and LC-MS/MS [155,156]. Metabolomics tools have been utilized to detect mycotoxin accumulation in different crops and food products [157,158,159].

The limit of quantification of different aflatoxins has been analyzed in different food samples, although aflatoxin levels may vary with the substrate on which the fungus is growing. The limit of detection for aflatoxin B1 and aflatoxin B2 is 3.0 µg/kg and 10.0 µg/kg, respectively. For aflatoxin G1, aflatoxin G2, and aflatoxin M1, the detection limit is around 10.0 µg/kg. Ochratoxin A and B’s detection levels are 15.0 µg/kg and 9.9 µg/kg, respectively [160].

The first genomic analysis of *Aspergillus flavus* identified more than 7000 unique Expressed Sequence Tags (EST) [161], and, subsequently, the functional genomes of the scale of ~12,000 were identified [162]. These bioinformatics tools deciphered the gene sequences responsible for the production of aflatoxin [163], and the presence of these genes in a particular sample can be determined by microarray analysis, quantitative reverse transcriptase (qRT-PCR) etc. [164,165,166]. Furthermore, around 240 different *A. flavus* strains were isolated from peanut seeds, and genome sequencing of all these strains was performed by next-generation sequencing analysis. The isolated strains were distributed into nine clades, and, out of them, three clades were non-aflatoxigenic, five were aflatoxigenic, and one belonged to *A.* *parasiticus* [167].

Besides genomics, the transcriptomics analysis is also imperative to analyze if the genes produce the relevant enzymes or not [168,169]. For transcriptome quantification, high-throughput tools, such as transcriptome shotgun sequencing (WTSS) and microarrays, are used [170,171,172,173]. For detecting mycotoxins, apart from microarrays, other high-throughput tools used were RT-qPCR and RNA-seq [174]. Transcriptome analysis of the fungus gives information about the interaction and relationship between the fungus and the host organism. In one of the studies, *Aspergillus flavus* isolated from *Zea mays* was put to RNA sequencing, which revealed the interaction and relationship between the fungus and the plant and helped build the interactome of the host and pathogen with mycotoxin production [175,176]. The presence of secondary metabolites also helped to develop detection systems [177]. Studies of different toxins explain that transcriptomic studies do not clarify the modes of actions of the toxins, so, classical toxicology and omics studies that explain the modes of action should also be performed [178,179]. It is important to mention that these studies of omics and metabolomics could help to detect the levels of infection in any food product with some limitations.

Moreover, another technique, the PCR reaction, for detecting fungal aflatoxins is not very specific as the structure of these toxins is complex and requires multiple genes for this purpose. The structural genes involved in the PCR are often those that also express other toxins (sterigmatocystin) produced by *A. versicolor* and *A. nidulans.* The DNA target region that can be used instead to identify the aflatoxin is the ribosomal DNA, such as internal transcribed spacer regions 1 and 2. Other DNA target regions include the 28S ribosomal DNA, majorly its 5′-end. The genes that are promisingly utilized for the detection of aflatoxins are *nor-1*, *omt-A*, and *ver-1* [180].

Another technique that can be used to detect is loop-mediated isothermal amplification (LAMP) [181], and one of the significant advantages of LAMP assays is that it is unaffected by inhibitors from the growth media or food matrix [182]. LAMP involves four primers that bind specifically to DNA and helps in better amplification of the DNA with enhanced reaction speed [183]. Six different binding sites enable specific amplification of target DNA. PCR and LAMP assays are species-specific and pose certain challenges for the detection of minor species. The *nor-1* gene-specific LAMP assay was utilized for the detection of certain species, and, utilizing it for 128 fungal species of 28 genera, synonyms of *A. flavus* and *A. parasiticus* were discovered, which were aflatoxigenic in nature [184]. Positive reactions are detected using neutral red during daylight to avoid unambiguity. The conidia of *Aspergillus parasiticus* was detected with a limit of ~210 conidia per reaction. The samples of nuts, dried figs, rice, spices, and raisins have been analyzed for the presence of aflatoxinogenic species in it. Detection of bacteria, protists, viruses, fungi, plants, and animals was also performed using the LAMP assay [185,186,187]. *Aspergillus* spp. producing aflatoxin was detected by developing a specific LAMP assay [188,189,190]. Rice samples were also analyzed, and it was found that rice can act as a relevant source of aflatoxin contamination [184,191]. Campylobacter in poultry carcasses has been detected using the LAMP method [192,193]. Mycotoxin contamination was detected in wheat grains using strategies of multiplex PCR and LC/MS/MS. Out of 34 samples assessed for mycotoxin contamination, many samples were found contaminated with *Fusarium* and *Aspergillus* species. The mycotoxins commonly found in food samples include aflatoxin B1, deoxynivalenol, and fumonisins [194].

### 3.6. Elisa-Based Detection

ELISA is another tool to detect the presence of pesticides, as well as food-borne pathogens. Carbaryl pesticide was analyzed in water and soil samples using ELISA.ELISA was also applied in case of detection of carbaryl pesticide in grains. The carbaryl present in some products, such as almonds, sweet potato, and peaches, is very low, and the sensitivity of this method is 460–1150 µg kg^−1^. The ELISA methods that were used were competitive homologous and heterologous ELISA methods. A heterologous CD-ELISA was used to detect carbyl, and sensitivity was increased by 12-fold compared to the homologous ELISA method. This method was applied in multiple food sources, and the sensitivity increased by ~75-fold. Furthermore, a 10-fold improved detection of carbaryl in food samples was performed using ELISA, coupled with chemiluminescence (ECL) [195].

Carbaryl pesticide present in rice samples can be detected using a capillary electrophoresis-based competitive immunoassay (CEIA), coupled with a detector of laser-induced fluorescence (LIF). The use of this method has enhanced the equilibrium and reduced the detection time within 8 min. CEIA can be coupled to ELISA, and, using the CEIA-ELISA method, the detection limit of carbaryl was found to be 0.05 ng/mL [196], and, comparing the CEIA-ELISA versus CEIA-LIF, the sensitivity of ELISA was 14 times less. Determination of the amount of carbaryl in spiked rice samples was performed with a simple pretreatment. The spiked rice samples were tested for recovery of carbaryl by using the CEIA-LIF detector [197].

Organophosphorus pesticides (OPs) can also be detected using the modern methods of ELISA. A competitive impedimetric immunoassay technique was developed for the detection of chlorpyrifos. This assay utilizes the particular affinity of immunoassay, along with an enzyme-based biocatalytic precipitation amplification approach. The Electro-deposited nanogold surface was modified with the help of a glassy carbon electrode. The chlorpyrifos antibody was anchored onto the modified electrode by gold-NH_2_ bond and gold-SH bonds. The reactivity of the electrode was improved by anchoring the appropriate concentration of antibody against chlorpyrifos. HRP (horseradish peroxidase) enzyme and bovine serum albumin-chlorpyrifos (BSA-CPF) were made to react with gold nanoparticles to yield the analyte competitor HRP-AuNP-BSA-CPF. Competitive immunoassay occurred between chlorpyrifos and HRP-AuNP-BSA-CPF to react with the CPF antibody. The immunoassay has been utilized to detect chlorpyrifos in vegetable samples (Chinese cabbage, Lettuce) [120]. The analysis of acetylcholinesterase electrochemical biosensor cannot estimate low concentrations of organophosphate pesticides present in food samples, such as vegetables and fruits, drinking water, and soil samples. The method is not sensitive and selective enough [198].

*Salmonella* in animal samples, such as pork, beef, and chicken, can be detected using a sandwich immunosensor assay [199]. For the *Escherichia coli* O157:H7 strain, rapid detection was developed where the enzyme-antibody conjugate mixture was used to label the cells. These labeled cells were taken on a 0.2 μm filter, and then the filter was placed on the electrode to measure the enzyme-substrate interaction [200,201]. Another immunosensor based on amperometric was developed [202]. It was based on the activity of β-galactosidase, and coliform bacteria were analyzed. Disposable screen-printed electrodes were used for simultaneous analysis of multiple samples [202]. In this case, the specificity was obtained by using the electrodes that are coated with the antibody specific for a particular bacterium. Different bacterial strains were optimized and analyzed, along with bacteriophages, and the enzymes that were analyzed include p-aminophenyl-α-D-glucopyranoside (pAP-α-GLU) for *B. cereus* and pAP-β-D-GLU(p-AP-β-GLU) for *Mycobacterium smegmatis* [148,149].

### 3.7. Microextraction and Chromatographic Techniques

Many different chromatographic techniques, such as TLC and HPTLC, have been employed to analyze diverse contamination in food products. *Aspergillus flavus* releases aflatoxins, which contaminate nuts, rice, beans, barley, food sources for fishes, etc. Aflatoxins can be detected by a series of steps, including sampling, purification, and concentration of the extract, followed by TLC and HPTLC. For the quantitative and qualitative detection of certain selected pesticides in wastewater and lake; RP-HPTLC and NP-TLC Gas chromatography-mass spectroscopy (GCMS) was utilized. A dispersive liquid-liquid microextraction method was sensitized for the same purpose. The disperser and extraction solvent were optimized by applying a univariate approach and box-behnken design was incorporated to analyze elements. The results described that the method was accurate and could be applied to a wide variety of samples [203].

Gas chromatography and liquid chromatography have been employed for detecting organic and inorganic contaminants. [204,205]. Besides this, different compounds have been quantitated using gas chromatography-mass spectrometry (GC-MS) [206]. Different techniques, such as solidified floating organic drop microextraction (SFODME) [186,207], switchable solvent liquid-phase microextraction (SS-LPME) [208], dispersive solid-phase microextraction based on magnetic nanoparticles (d-SPE-MNP) [209], hollow fiber liquid-phase microextraction (HF-LPME) [210], single drop microextraction (SDME) [211], and solid-phase microextraction (SPME) [212], have been utilized for enhancement of detection power. Rezaee et al. devised a method to extract polycyclic aromatic compounds by dispersive liquid-liquid microextraction (DLLME) [213]. This method is more accurate and can be used to detect contaminants at very low concentrations [214]. Certain microextraction techniques and DLLME have utilized non-toxic chemicals, making the procedure green and environmentally friendly [215]. 

### 3.8. Biosensors

Biosensors are the analytical devices that are utilized for the estimation of chemical and biological analytes. The main components of the biosensor include the detector to detect the analyte, which also acts as a signal generator, signal transducer and a reading and amplifying device. Biosensors can be classified into many types depending on the contaminant they detect, the detector system, and the transducer system they possess.

#### 3.8.1. Nanobiosensor

Nanobiosensors are biosensors that have nano-scale entities attached with the tranducer of the biosensor. Previously, techniques, such as Surface Enhanced Raman Spectroscopy (SERS), have been used to detect contaminants in many food samples, fishes, and melamine in milk. This technique was then modified to synthesize standing AuNR arrays. Introduction of standing AuNR arrays induced a potent electromagnetic field, but it made the system capable of analyzing milk, orange juice, and grapefruit juice for carbaryl. Contaminants can be detected as low as 50 ppb by this modified method, which can be used in different food samples [216]. Magnetic nanoparticles can also be used for detecting various analytes. These have been thoroughly studied and have a wide range of applications ranging from functioning as a glucose sensor [217], for quantified estimation and removal of rhodamine [218], for the diagnosis of malaria [219], and for enzyme immune assay atrazine sensor [220,221].

#### 3.8.2. DNA Biosensor

The DNA biosensor is based on the concept that a specific nucleotide sequence called a probe is immobilized onto a chosen transducer based on its complementarity bind and detects specific nucleic acid sequence in a sample. Detection of specific nucleic acid sequences is initiated by a hybridization reaction between the probe and nucleic acid in a sample. DNA hybridization can be detected using electrochemical transducers, which are more robust and sensitive [222,223,224,225,226,227,228]. To increase the efficacy of the detection, DNA biosensors have been coupled to PCR. Different pathogenic bacteria can be detected simultaneously by using a disposable electrochemical low-density genosensor array [223,226,227,228].

In another study, a screen-printed array of gold electrodes, which were modified using thiol-tethered single-strand DNA probes, was used for detecting bacteria present in different samples [80]. The surface-tethered and biotinylated signaling probes were bound to the samples containing the bacteria of interest by the sandwich hybridization technique. The hybrids formed were bound to a streptavidin–alkaline phosphatase conjugate. The conjugate was further exposed to an α-naphthyl phosphate solution, and the signal generated was detected by differential pulse voltammetry. These systems can further be improved to develop a strain-specific assay in which the probe can be a sequence of a specific gene encoding a toxin produced by the bacterial strain. This would help to differentiate if the contamination is toxic or not. 

On the same lines, target oligonucleotides can be immobilized onto carbon paste electrode, and the hybridization can be detected by chronopotentiometry. These electrodes were utilized for simultaneous and fast analysis of *Lysteria monocytogenes*, *Cryptosporidium*, *Salmonella enterica*, *S. aureus*, *Giardiaspp*, *E. coli* 0157:H7, and *Mycobacterium tuberculosis*. The electrodes have been coupled with primers with magnetic moieties for the electrochemical detection of multiple pathogens in food samples [229]. In another attempt, a DNA target hybridized to both biotinylated capture and digoxienin probe was used as a sensor. It was further attached to streptavidin-modified magnetic beads and was isolated using a graphite-epoxy composite-based magneto electrode system. Anti-digoxigenin horseradish peroxidase (HRP) was used as the electrochemical detector. PCR- amplified DNA samples were also estimated using this method. The assay has been tested for *Salmonella* spp. [230]. Interdigitated gold array electrodes (IDA electrodes) were also utilized for the estimation of different compounds very sensitively. The IDA electrode lies in the nanometer range, and capture probes immobilized on it were thiol-modified oligonucleotides. RNA hybridization can be improved by adding three additional molecules in adjacent proximity to the place of interaction. The RNA bound to the electrode can be hybridized with a biotin-labeled detector oligonucleotide, enabling the binding of the conjugates of avidin-linked alkaline phosphatase. A multi-potentiostat detected the electrical signal generated. The sensitivity of these systems can be enhanced by 60% by changing the hybridization patterns. PCR-free methods have also been devised for the analysis of food contaminants, and, in this regard, an RNA-biosensor was devised for detection of *E. coli* in water samples, which makes this technology rapid, specific, and sensitive [231].

In a separate study, an isothermal NASBA technique was used to estimate, prepare, and amplify the sample. A DNA/RNA-based biosensor was utilized for the estimation of the amplified RNA. The samples can be detected as low as 5 fmol per sample and 40 *E. coli* CFU mL^−1^ [80].

### 3.9. Smartphone-Based Biosensing

Looking at the increased demand of technology and the latest technological advancements, smartphones have become an inevitable part of our lives. They have built in sensors, better connectivity, portability, and operability. Food evaluation can be made easy and widely available by linking the biosensors with smartphones. These user-friendly and portable detectors are capable of detecting toxins, allergens, contaminants, and pathogens. Food products get contaminated during the entire processing, retail, storage, and consumption protocol [232,233]. Smartphone-based biosensors have replicated the conventional methods of detection but are more powerful [234,235,236,237,238].

Biosensors have a very high potential for detecting pathogens in food samples. Biosensors are also compatible with portable devices and are, therefore, easily incorporated into portable devices for the detection of food contaminants [232,235,239,240]. The latest operating systems, sensors, transducers, and data processors have enabled smartphones to act as excellent data processors [235,241,242]. In healthcare diagnosis, smartphones have been utilized for particular colorimetric and fluorescence assays. Smartphones have also been utilized for the evaluation of food quality and environmental monitoring [243,244,245]. By 2018, 67% of the global population utilized mobile services [246]. On-site sensing devices and systems have changed drastically by the introduction of smartphones at this front. 

#### 3.9.1. Smartphone-Based Optical Biosensors

**Figure 6:**
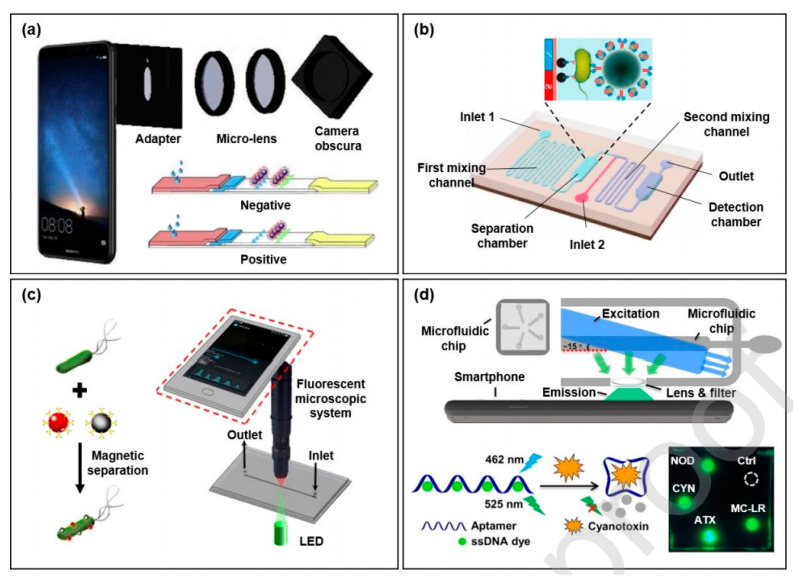
(**a**) 3D design of the solid phase latex microsphere immunochromatography platform (SIAP) in the smartphone for detecting the presence of zearalenone in cereals and feed. Obtained with permission from Reference [247], copyright (2018) Elsevier (License number 5184930674381). (**b**) Colorimetric biosensor for detecting *E. coli* O15:H7 adapted from Reference [248] with permission from copyright (2019) Elsevier (License number 5184910590426). (**c**) *Salmonella typhimurium* detection adapted from Reference [250] with permission from copyright (2019) Elsevier (License number 5184910825826). (**d**) Multiple compound detection using fluorescent aptasensor adapted with permission from Reference [251], copyright (2019) American Chemical Society.

Smartphones can be made compatible with multiple biosensors for a wide range of applications. Smartphones combined with optical biosensors, such as colorimetry, fluorescence, etc., can be utilized for real-time food analysis. 3D design of the solid phase latex microsphere immunochromatography platform (SIAP) in the smartphone for detecting the presence of zearalenone in cereals and feed has been utilized [247]. One example of food analysis is the microfluidic biosensor designed for *E. coli* O157:H7 [243,244,247,248]. In this microfluidic biosensor, gold nanoparticles were analyzed for their aggregation owing to *E. coli* in the samples (Figure 6b) [249]. Magnetic nanoparticles with specific antibodies can also be used to analyte separation, density, and detection of food pathogens [244,247,250]. Another biosensor was also developed for the presence of *Salmonella typhimurium*. The detection was performed using a fluorescent detector (Figure 6c) [251].

Similarly, a multichannel fluorescence detector was utilized to detect four types of cyanotoxins (Figure 6d) [252]. Aptamer-based dye assay is used in the multichannel detector to generate fluorescent or colorimetric responses. Different light sources excite the fluorophores, and the change in the fluorescence can be detected by the smartphone camera [244,251,253].

#### 3.9.2. Smartphone-Based Electrochemical Biosensor

**Figure 7:**
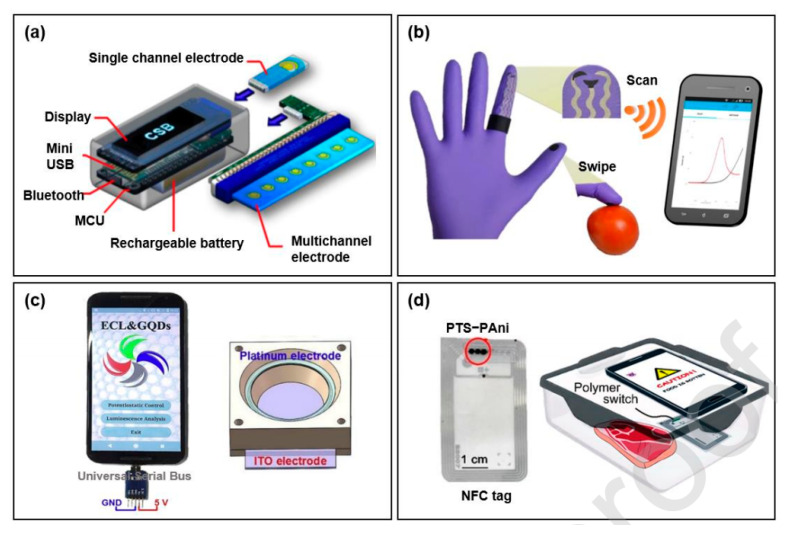
Evaluation of food samples using smartphone-based electrochemical biosensors. (**a**) Pocket size detector. Adopted from Reference [257] with permission from the American Chemical Society. (**b**) Gloves compatible with Smartphone-based biosensor, adapted from Reference [260] with permission from American Chemical Society. (**c**) Electrochemiluminescence system based on smartphones for *Escherichia coli* detection, adapted from Reference [241] with permission from Elsevier. Adapted with permission from Elsevier (License Number 5184940510188). (**d**) Food spoilage detection by a wireless badge, adapted from Reference [232] with permission American Chemical Society, 2021.

Electrochemical biosensors have also been incorporated into smartphones apart from optical biosensors. Smartphones have been incorporated with electrochemical techniques, including amperometric [254,255], potentiometric [240], and impedimetric method [256,257]. Integrated exogenous antigen testing (iEAT) has been initiated for on-site detection of food allergens [258] (Figure 6 and Figure 7). 

The components involved were smartphone, electronic reader, mini display screen, microcontroller unit (MCU) for signal processing, rechargeable battery, Bluetooth communications module, c card-edge connector, and a disposable allergen extraction kit. It includes a disposable allergen extraction device, electronic reader, and application for smart phone. Allergens are captured through immunogenic enrichment, and a signal is generated in the presence of HRP and a chromogenic electron mediator (Figure 7a). The biomedical analysis involved using a smartphone-based electrochemiluminescence system that linked optical analysis with electrochemical excitation [242,255]. Serial bus-based and camera-based imaging methods were involved (Figure 7c) [242]. The detection of *E. coli* was performed by using platinum and indium tin oxide electrodes [241]. 

Chloropyrifos, malathion, and diazinon can be detected using fluorophore-quencher nano-pairs, coupled with fluorescent aptamer-based lateral flow biosensor (apta-LFB) [259,260,261]. In the lateral flow biosensor, instead of antibodies, aptamers have been used as recognition elements. They exhibit better specificity and stability as compared to the biosensor utilizing antibodies. Detection limit of malathion and chlorpyrifos was in the range of ~0.7 ng/mL, and, for diazinon, it was around 6.7 ng/ mL [262]. 

### 3.10. DNA Microarray

The obsolete and slow detection methods have been surpassed by the advancement and introduction of DNA microarrays. DNA microarrays have enabled the detection of pathogens in multiple samples in a single go, improving the efficacy, specificity, and time of detection.

Microarray technology has enhanced the specificity by incorporating multiple specific probes; thus, the false positives often resulting from the cross-contamination of microorganisms can be eliminated [263]. Gene chip arrays have been designed for studying the food contaminants by targeting virulent pathogenic genes [264,265,266].

A large number of genes can be analyzed quantitatively at the same time. It has the benefits of accuracy and rapid bioanalysis at low cost. Various types of microarray have been synthesized, but, majorly, they can be divided into the following types [193]:

1. Longer probe length and increased specificity chip was designed by Stanford University, but it has a disadvantage of low chip density.
2. In-situ synthesis technology produces chips by photolithography with probes of only 25 mer length. Multiple probes have been used to avoid misjudgment for a single gene.
3. Micro bead placement method of microarray preparation where nucleic acid probes are put loaded on micro particles on a particular slide.
4. qPCR array where RT-PCR primer and probe were synthesized in well plates microfluidic disk, and the detection was carried out by quantitative PCR.

In one example, the toxicity due to *Shiga* toxin produced by *E. coli* (STEC) O104: H7 and the toxicity produced by *Salmonella* strains isolated animal products were analyzed by DNA microarray [267,268].

## 4. Conclusions

Spoilage of food results in food insecurity around different regions of the world, leading to huge economic losses both all the people involved in the chain, starting from the producers to the consumers. Diverse methods and strategies have been used for the detection of contaminants in food samples. Some of these are PCR-based, either simplex or multiplex, with some using a real-time format, microextraction and chromatographic techniques, and omic tools biosensors based on DNA or nanotechnology. Techniques involving biosensors are reliable, safe, and specific, and they can be used during food manufacturing processes to monitor the inline processes. Spectroscopic techniques provide accurate analysis of the chemical contaminants in food, whereas techniques involving detection of molecules, such as DNA and proteins, include biosensors. The latest technologies, such as using smartphone-based biosensors for the detection of contaminants, have a lot to offer, and the research on food spoilage will take a different course when these technologies are available widely to the masses.

## Figures and Tables

**Table 1:** Types of contaminants in food.

Organism/Chemical	Name	Food-Borne Diseases and Problems	High-Risk Foods
Gram-Positive bacteria	*Listeria monocytogenes*	Food borne-listeriosis; Diarrhea	Meat-related products (Deli or ready-to-consume),such ascold smoked-fishery items, meat, sausages, etc.
	*Bacillus cereus*	Emetic and diarrheal syndrome	Pasteurized milk and dairy products, red meat, beef, lamb, vension
	*Bacillus licheniformis*, *B. coagulans*, *Geobacillus stearothermophilus*, *Clostridium algidixylanolyticum*, *C. algidicarnis*, *C. gasigenes*, *C. frigidicarnis* and *C. estertheticum*	Inflammatory bowel disease, Crohn’s disease	Dry milk, and tomato juice (low-acid)
	*Lactobacillus lactis*, *Leuconostoc* spp.	Diarrhea, wounds and urinary tract infection, bacteremia, pneumonia, and cerebral hemorrhage	Fermented food and beverages, wine, beer, and fruit juices, vacuum packaged meat, fish, and poultry products
	*Staphylococcus aureus*	Suppurative infection, septicemia, pneumonia, sepsis, pericarditis, pseudomembranous colitis	Meat, milk, fish and their products, eggs, and cold food savory
	*Clostridium botulinum*	Respiratory and muscle relaxation paralysis, botulism, blurred vision	Cured meat and Canned products
Gram-Negative bacteria	*Pseudomonas*	Cystic fibrosis, respiratory and urinary infections, pneumonia as hospital-acquired disease	Vegetables and fruits, red meat, poultry, fish, milk, and milk products
	*Enterobacteriaceae*	Diarrheal disease, septicemia; bacteremias, respiratory disease; wound and burn infections; urinary tract infections; and meningitis due to its pathogenicity	Raw meat, chicken and beef, fresh cream desserts
	*Salmonella typhimurium*	Stomach pain, typhoid fever, diarrhea, nausea, headache, gastroenteritis, fever, chills, septicemia	Raw forms ofdairy produce, egg, raw or less cookedmeat, poultry, and seafood. Unprocessedsalads and chocolate.
	*Escherichia coli*	Nausea, diarrhea, stomach pain, fever, headache, and chills	Raw forms of dairy products, raw or less cooked meat, poultry products, such asegg, and seafood
	*Campylobacter*	Nausea, Diarrhea, Stomach pain, fever, and headache	Raw milk, raw or undercooked meat and poultry
	*Shigella*	Bacterial dysentery	Raw and cooked food
	*Cronobacter*	Neonatal meningitis, necrotizing colitis and bacteremia	Milk powder and infant feed
Fungus	*Aspergillus*, *Fusarium*,and *Penicillium*	Athlete’s foot, ringworm, aspergillosis, histoplasmosis and coccidiodomycosis	Fresh seafood, packaged meats, delicatessen salads
Parasite	Trematode (*Opisthorchisspp*; *Clonorchisspp*; *Paragonimusspp*; *Fasciolaspp*)	Trematodiases, Clonorchiasis, fascioliasis, opisthorchiasis, Paragonimiasis, severe lung and liver problem; fever; nausea	Infected raw vegetables, aquatic vegetables, raw fish or raw meat of animals feeding on these, crabs
	*Toxoplasma gondii*	Toxoplasmosis	Beef, pork, shellfish, fruits, vegetables
	*Giardia lamblia*	Giardiasis	Shellfish
	*Entamoeba histolytica*	Acute dysentery, Ameboma, perianal ulceration	Raw fruits and vegetables
	*Trypanosoma cruzi*	Chagas disease	Raw fruits and vegetables
Viruses	Hepatitis A virus	Fever, malaise, anorexia, nausea, jaundice	Vegetables, fruits, shellfish, iced drinks, milk, and dairy produce
	Norovirus	Diarrhea, vomiting, nausea, muscle and stomach cramps	Contaminated drinking water, raw salads, raw shellfish or oysters, berries, and frozen food products
Heavy metals	Arsenic	Lung and bladder diseases, skin infections, heart disorders	Contaminated drinking water, cereals, vegetables
Pesticides	Chlorpyrifos	Neuromuscular disorders, nausea, headache, acute poisoning	Contaminated farm produce
	Carbaryl pesticide	Reproductive and developmental toxicity, cholinesterase inhibition, intestinal agenesis	Contaminated farm produce

## Data Availability

The data presented in this study are available on request from the corresponding author.

## References

[1] Behera B.K., Rout P.K., Behera S. (2019). Move towards Zero Hunger.

[2] Bain L.E., Awah P.K., Geraldine N., Kindong N.P., Siga Y., Bernard N., Tanjeko A.T. (2013). Malnutrition in Sub–Saharan Africa: Burden, causes and prospects. Pan Afr. Med. J..

[3] World Health Organization (2020). The State of Food Security and Nutrition in the World 2020: Transforming Food Systems for Affordable Healthy Diets.

[4] Gram L., Ravn L., Rasch M., Bruhn J.B., Christensen A.B., Givskov M. (2002). Food spoilage—Interactions between food spoilage bacteria. Int. J. Food Microbiol..

[5] Odeyemi O.A., Alegbeleye O.O., Strateva M., Stratev D. (2020). Understanding spoilage microbial community and spoilage mechanisms in foods of animal origin. Compr. Rev. Food Sci. Food Saf..

[6] Gustavsson J., Cederberg C., Sonesson U., Van Otterdijk R., Meybeck A. (2011). Global Food Losses and Food Waste-FAO Report.

[7] Cao X. (2020). COVID-19: Immunopathology and its implications for therapy. Nat. Rev. Immunol..

[8] Chatterjee A., Abraham J. (2018). Microbial Contamination and Food Degradation.

[9] Dogan B., Boor J. (2003). Genetic diversity and spoilage potentials among Pseudomonas from fluid milk products and dairy processing plants. Appl. Environ. Microbiol..

[10] Raposo A., Pérez E., de Faria C.T., Ferrús M.A., Carrascosa C. (2017). Food Borne Pathogens and Antibiotic Resistance.

[11] Dainty H., Mackey M. (1992). The relationship between the phenotypic properties of bacteria from chill-stored meat and spoilage processes. J. Appl. Bacteriol..

[12] Walker S.J., Archer P., Banks J.G. (1989). Growth of Pathogenic Bacteria at Chill Temperatures.

[13] Elliot E.L., Kaysner C.A., Jackson L., Tamplin M.L., Merker R.L. (1998). *V. cholerae*, *V. parahaemolyticus*, *V. vulnificus*, and other *Vibrio* spp.. Food and Drug Administration Bacteriological Analytical Manual.

[14] Borch E., Kant-Muermans M., Blixt Y. (1996). Bacterial spoilage of meat and cured meat products. Int. J. Food Microbiol..

[15] Arslan S., Eyi A., Ozdemir F. (2011). Spoilage potentials and antimicrobial resistance of Pseudomonas spp. isolated from cheeses. J. Dairy Sci..

[16] Molina G., Pimentel M., Pastore G. (2013). *Pseudomonas*: A promising biocatalyst for the bioconversion of terpenes. Appl. Microbiol. Biotechnol..

[17] American Thoracic Society (2005). Guidelines for the management of adults with hospital-acquired, ventilator-associated, and healthcare-associated pneumonia. Am. J. Resp. Crit. Care Med..

[18] Gaynes R., Edwards J. (2005). Overview of nosocomial infections caused by gram-negative bacilli. Clin. Infect. Dis..

[19] Kolleff H., Shorr A., Tabak Y., Gupta V., Liu L., Johannes R. (2005). Epidemiology and outcomes of health-care associated pneumonia: Results from a large US database of culture-positive pneumonia. Chest.

[20] Brenner D.J., Farmer J.J. (2015). Bergey’s Manual of Systematics of Archaea and Bacteria.

[21] Silvetti T., Morandi S., Brasca M. (2018). Growth factors affecting gas production and reduction potential of vegetative cell and spore inocula of dairy-related Clostridium species. LWT.

[22] Cousin M.A. (1982). Presence and activity of psychotrophic microorganisms in milk and dairy products: A review. J. Food Prot..

[23] Walker S.J. (1988). Major spoilage organisms in milk and dairy products. J. Soc. Dairy Technol..

[24] Podrzaj L., Burtscher J., Küller F., Domig K.J. (2020). Strain-Dependent Cheese Spoilage Potential of Clostridium tyrobutyricum. Microorganisms.

[25] Clark F.M., Dehr A. (1946). A study of butyric acid-producing anaerobes isolated from spoiled canned tomatoes. Food Res..

[26] Townsend C.T. (1939). Spore-forming anaerobes causing spoilage in acid canned foods. Food Res..

[27] Spiegelberg C.H. (1940). *Clostridium pasteurianum* associated with spoilage of an acid canned fruit. Food Res..

[28] Feng G., Churey J.J., Worobo R.W. (2010). Thermoaciduric Clostridium pasteurianum spoilage of shelf-stable apple juice. J. Food Prot..

[29] Jensen T.Ø., Kvist T., Mikkelsen M.J., Christensen P.V., Westermann P. (2012). Fermentation of crude glycerol from biodiesel production by Clostridium pasteurianum. J. Ind. Microbiol. Biotechnol..

[30] Schwarz K.M., Grosse-Honebrink A., Derecka K., Rotta C., Zhang Y., Minton N.P. (2017). Towards improved butanol production through targeted genetic modification of Clostridium pasteurianum. Metab. Eng..

[31] Sangeetha R., Karunanithi T. (2011). Response Surface Methodological Analysis on Growth of *Clostridium pasteurianum*. Int. J. Chem. Technol. Res..

[32] Broda D.M., Saul D.J., Lawson P.A., Bell R.G., Musgrave D.R. (2000). Clostridium gasigenes sp. nov., a psychrophile causing spoilage of vacuum-packed meat. Int. J. Syst. Evol. Microbiol..

[33] Lawson P., Dainty R.H., Kristiansen N., Berg J., Collins M.D. (1994). Characterization of a psychrotrophic Clostridium causing spoilage in vacuum-packed cooked pork: Description of *Clostridium algidicarnis* sp. nov. Lett. Appl. Microbiol..

[34] Dainty R.H., Edwards R.A., Hibbard C.M. (1989). Spoilage of vacuum-packed beef by a *clostridium* sp.. J. Sci. Food Agric..

[35] Collins M.D., Rodrigues U.M., Dainty R.H., Edwards R.A., Roberts T.A. (1992). Taxonomic studies on a phsychrophilic *Clostridium* from vacuum-packed beef: Description of *Clostridium estertheticum* sp.. FEMS Microbiol. Lett..

[36] Adam K.H., Flint S.H., Brightwell G. (2010). Psychrophilic and psychrotrophic clostridia: Sporulation and germination processes and their role in the spoilage of chilled, vacuum-packaged beef, lamb and venison. Int. J. Food Sci. Technol..

[37] Coghill D., Juff H.S. (1979). Incidence of psychotrophic spore forming bacteria in pasteurized milk and cream products and effect of temperature on their growth. Aust. J. Dairy Technol..

[38] Despina K.V. (1992). Biochemical activities of *Bacillus* species isolated from flat sour evaporated milk. J. Dairy Sci..

[39] Scheldeman P., Herman L., Foster S., Heyndrickx M. (2006). Bacillus sporothermodurans and other highly heat-resistant spore formers in milk. J. Appl. Microbiol..

[40] Crielly E., Logan N., Anderton A. (1994). Studies on the *Bacillus flora* of milk and milk products. J. Appl. Bacteriol..

[41] Dhakal R., Seale R.B., Deeth H.C., Craven H., Turner M.S. (2014). Draft genome comparison of representatives of the three dominant genotype groups of dairy *Bacillus licheniformis* strains. Appl. Environ. Microbiol..

[42] Jonghe V.D., Coorevits A., Block J.D., Coillie E.V., Grijspeerdt K., Herman L., Vos P.D., Heyndrickx M. (2010). Toxinogenic and spoilage potential of aerobic spore-formers isolated from raw milk. Int. J. Food Microbiol..

[43] Reginensi S.M., Gonzalez M.J., Olivera J.A., Sosa M., Juliano P., Bermudez J. (2011). RAPD-based screening for spore-forming bacterial populations in Uruguayan commercial powdered milk. Int. J. Food Microbiol..

[44] Hammer P., Lembke F., Suhren G., Heeschen W. (1995). Characterization of a heat resistant mesophilic *Bacillus* species affecting quality of UHT-milk: A preliminary report. Kiel. Milchwirtsch. Forschungsber..

[45] Klijn N., Herman L., Langeveld L., Vaerewijck M., Wagendorp A.A., Huemer I., Weerkamp A.H. (1997). Genotypical and phenotypical characterization of *Bacillus sporothermodurans* strains, surviving UHT sterilisation. Int. Dairy J..

[46] Gopal N., Hill C., Ross P.R., Beresford T.P., Fenelon M.A., Cotter P.D. (2015). The prevalence and control of Bacillus and related spore-forming bacteria in the dairy industry. Front. Microbiol..

[47] Veld H.I., Jos H.J. (1996). Microbial and biochemical spoilage of foods: An overview. Int. J. Food Microbiol..

[48] Khalid K. (2011). An overview of lactic acid bacteria. Int. J. Biosci..

[49] Xu Z., Luo Y., Mao Y., Peng R., Chen J., Soteyome T., Bai C., Chen L., Liang Y., Su J. (2020). Spoilage lactic acid bacteria in the brewing industry. J. Microbiol. Biotechnol..

[50] Buu-Hoi A., Branger C., Acar J.F. (1985). Vancomycin-resistant streptococci or *Leuconostoc* sp.. Antimicrob. Agents Chemother..

[51] Aguirre M., Collins M.D. (1993). Lactic acid bacteria and human clinical infection. J. Appl. Bacteriol..

[52] Miller M.W. (1979). Yeasts in food spoilage: An update. Food Technol..

[53] dos Santos Couto M.M.B. (1995). Development and Implementation of Molecular Typing Techniques for Identification of Food Spoilage Yeasts. Ph.D. Thesis.

[54] Fowler J.L., Clark. W.S. (1975). Microbiology of delicatessen salads. J. Milk Food Technol..

[55] Koburger J.A. (1971). Fungi in foods. II. Some observations on acidulants used to adjust media pH for yeasts and mold counts. J. Milk Food Technol..

[56] Koburger J.A. (1972). Fungi in foods. III. The enumeration of lipolytic and proteolytic organisms. J. Milk Food Technol..

[57] Winter F.H., York G.K., El-Nakhal H. (1971). Quick counting method for estimating the number of viable microbes on food and food processin equipment. Appl. Microbiol..

[58] Dagnas S., Membré J.M. (2013). Predicting and preventing mold spoilage of food products. J. Food Prot..

[59] Aiko V., Mehta A. (2015). Occurrence, detection and detoxification of mycotoxins. J. Biosci..

[60] Liu B.H., Yu F.Y., Wu T.S., Li S.Y., Su M.C., Wang M.C., Shih S.M. (2003). Evaluation of genotoxic risk and oxidative DNA damage in mammalian cells exposed to mycotoxins, patulin and citrinin. Toxicol. Appl. Pharmacol..

[61] Adeyeye S.A.O. (2016). Fungal mycotoxins in foods: A review. Cogent Food Agric..

[62] Chauhan R., Singh J., Sachdev T., Basu T., Malhotra B.D. (2016). Recent advances in mycotoxins detection. Biosens. Bioelectron..

[63] Oliveira I.S., da Silva Junior A.G., de Andrade C.A.S., Oliveira M.D.L. (2019). Biosensors for early detection of fungi spoilage and toxigenic and mycotoxins in food. Curr. Opin. Food Sci..

[64] Hutton M., Hutchinson T.C., Meema K.M. (1987). Human health concerns of lead, mercury, cadmium and arsenic. Lead, Mercury, Cadmium and Arsenic in the Environment.

[65] Sharma B., Thakur S., Mamba G., Prateek, Gupta R.K., Gupta V.K., Thakur V.K. (2020). Titania Modified Gum Tragacanth Based Hydrogel Nanocomposite for Water Remediation. J. Environ. Chem. Eng..

[66] Abnet C. (2007). Carcinogenic food contaminants. Cancer Investig..

[67] Schantz S.L., Gardiner J.C., Gasior D.M., McCaffrey R.J., Sweeney A.M., Humphrey H.E. (2004). Much ado about something: The weight of evidence for PCB effects on neuropsychological function. Psychol. Sch..

[68] Alexander J., Benford D., Cockburn A., Cravedi J.P., Dogliotti E., Di Domenico A., Fernandez-Cruz M.L., Fink-Gremmels J., Furst P., Galli C. (2008). Polycyclic Aromatic Hydrocarbons in Food Scientifc Opinion of the Panel on Contaminants in the Food Chain. Food Saf. Auth. J..

[69] Li Q.Q., Loganath A., Chong Y.S., Tan J., Obbard J.P. (2006). Persistent organic pollutants and adverse health effects in humans. J. Toxicol. Environ. Health Part A.

[70] Ritter L., Solomon K.R., Forget J., Stemeroff M., O’leary C. (1995). A review of selected persistent organic pollutants for the International Programme on Chemical Safety (IPCS)..

[71] Mostafavi H.A., Fathollahi H., Motamedi F., Mirmajlessi S.M. (2010). Food irradiation: Applications, public acceptance and global trade. Afr. J. Biotechnol..

[72] Walker J.S., Don G.W. (2013). Mathematics and Music.

[73] Khan M.F., Wesley S.G. (2011). Assessment of health safety from ingestion of natural radionuclides in seafoods from a tropical coast, India. Mar. Pollut. Bull..

[74] Carvalho F.P., Oliveira J.M. (2010). Uranium isotopes in the Balkan’s environment and foods following the use of depleted uranium in the war. Environ. Int..

[75] Brennwald M.S., Van Dorp F. (2009). Radiological risk assessment and biosphere modelling for radioactive waste disposal in Switzerland. J. Environ. Radioact..

[76] Pröhl G., Olyslaegers G., Kanyar B., Pinedo P., Bergstrom U., Mobbs S., Eged K., Katona T., Simon I., Hallberg U.B. (2005). Development and comparison of five site-specifc biosphere models for safety assessment of radioactive waste disposal. J. Radiol. Prot..

[77] Thompson L.A., Darwish W.S. (2019). Environmental chemical contaminants in food: Review of a global problem. J. Toxicol..

[78] Jay J.M., Loessner M.J., Golden D.A. (2005). Introduction to foodborne pathogens. Modern Food Microbiology. Food Science Text Series.

[79] An X., Zuo P., Ye B.C. (2020). A single cell droplet microfluidic system for quantitative determination of food-borne pathogens. Talanta.

[80] Palchetti I., Mascini M. (2008). Electroanalytical biosensors and their potential for food pathogen and toxin detection. Anal. Bioanal. Chem..

[81] Zhu X.D., Shi X., Chu J., Ye B., Zuo P., Wang Y.H. (2018). Quantitative analysis of the growth of individual Bacillus coagulans cells by microdroplet technology. Biores. Bioprocess..

[82] Zhu X.D., Shi X., Wang S.W., Chu J., Zhu W.H., Ye B.C., Zuo P., Wang Y.H. (2019). High-throughput screening of high lactic acid-producing Bacillus coagulans by droplet microfluidic based flow cytometry with fluorescence activated cell sorting. RSC Adv..

[83] Bian X., Jing F., Li G., Fan X., Jia C., Zhou H., Jin Q., Zhao J. (2015). A microfluidic droplet digital PCR for simultaneous detection of pathogenic Escherichia coli O157 and Listeria monocytogenes. Biosens. Bioelectron..

[84] Jang M., Jeong S.W., Bae N.H., Song Y., Lee T.J., Lee M.K., Lee S.J., Lee K.G. (2017). Droplet-based digital PCR system for detection of single-cell level of foodborne pathogens. BioChip J..

[85] Oh J.H., Park M.K. (2016). Immunosensors combined with a light microscopic imaging system for rapid detection of Salmonella. Food Control.

[86] Srisa-Art M., Boehle K.E., Geiss B.J., Henry C.S. (2018). Highly Sensitive Detection of Salmonella typhimurium Using a Colorimetric Paper-Based Analytical Device Coupled with Immunomagnetic Separation. Anal. Chem..

[87] Scallan E., Hoekstra R.M., Angulo F.J., Tauxe R.V., Widdowson M.A., Roy S.L., Jones J.L., Griffin P.M. (2011). Foodborne illness acquired in the United States—Major pathogens. Emerg. Infect. Dis..

[88] Ahn S., Walt D.R. (2005). Detection of Salmonella spp. Using Microsphere-Based, Fiber-Optic DNA Microarrays. Anal. Chem..

[89] Jokerst J.C., Adkins J.A., Bisha B., Mentele M.M., Goodridge L.D., Henry C.S. (2012). Development of a paper-based analytical device for colorimetric detection of select foodborne pathogens. Anal. Chem..

[90] Xu X., Ma X., Wang H., Wang Z. (2018). Aptamer based SERS detection of Salmonella typhimurium using DNA-assembled gold nanodimers. Microchim. Acta.

[91] Martinez A.W., Phillips S.T., Butte M.J., Whitesides G.M. (2007). Patterned paper as a platform for inexpensive, low-volume, portable bioassays. Angew. Chem..

[92] Chen Y., Zilberman Y., Mostafalu P., Sonkusale S.R. (2015). Paper based platform for colorimetric sensing of dissolved NH3 and CO_2_. Biosens. Bioelectron..

[93] Barbosa A.I., Gehlot P., Sidapra K., Edwards A.D., Reis N.M. (2015). Portable smartphone quantitation of prostate specific antigen (PSA) in a fluoropolymer microfluidic device. Biosens. Bioelectron..

[94] Fan X.Y., Jia C.P., Yang J., Li G., Mao H.J., Jin Q.H., Zhao J.L. (2015). A microfluidic chip integrated with a high-density PDMS-based microfiltration membrane for rapid isolation and detection of circulating tumor cells. Biosens. Bioelectron..

[95] Martins D., Levicky R., Song Y.A. (2015). Enhancing the speed of morpholino-DNA biosensor by electrokinetic concentration of DNA in a microfluidic chip. Biosens. Bioelectron..

[96] Tan Y.F., Tang T.T., Xu H.S., Zhu C.Q., Cunningham B.T. (2015). High sensitivity automated multiplexed immunoassays using photonic crystal enhanced fluorescence microfluidic system. Biosens. Bioelectron..

[97] Phillips S.T., Lewis G.G. (2014). The expanding role of paper in point-of-care diagnostics. Expert Rev. Mol. Diagnostics.

[98] Gubala V., Harris L.F., Ricco A.J., Tan M.X., Williams D.E. (2012). Point of care diagnostics: Status and future. Anal. Chem..

[99] Warsinke A. (2009). Point-of-care testing of proteins. Anal. Bioanal. Chem..

[100] Peeling R.W., Holmes K.K., Mabey D., Ronald A. (2006). Rapid tests for sexually transmitted infections (STIs): The way forward. Sex. Transm. Infect..

[101] Hu J., Wang S.Q., Wang L., Li F., Pingguan-Murphy B., Lu T.J., Xu F. (2014). Advances in paper-based point-of-care diagnostics. Biosens. Bioelectron..

[102] Xia Y., Si J., Li Z. (2016). Fabrication techniques for microfluidic paper-based analytical devices and their applications for biological testing: A review. Biosens. Bioelectron..

[103] Li X., Ballerini D.R., Shen W. (2012). A perspective on paper-based microfluidics: Current status and future trends. Biomicrofluidics.

[104] Krishnan S., Kumar Narasimhan A., Gangodkar D., Dhanasekaran S., Kumar Jha N., Dua K., Thakur V.K., Kumar Gupta P. (2021). Aptameric Nanobiosensors for the Diagnosis of COVID-19: An Update. Mater. Lett..

[105] Anthony P.C., Perez C.F., García-García C., Block S.M. (2012). Folding energy landscape of the thiamine pyrophosphate riboswitch aptamer. Proc. Natl. Acad. Sci. USA.

[106] Frieda K.L., Block S.M. (2012). Direct observation of cotranscriptional folding in an adenine riboswitch. Science.

[107] Pourmadadi M., Shayeh J.S., Omidi M., Yazdian F., Alebouyeh M., Tayebi L. (2019). A glassy carbon electrode modified with reduced graphene oxide and gold nanoparticles for electrochemical aptasensing of lipopolysaccharides from Escherichia coli bacteria. Microchim. Acta.

[108] Xu W., Tian J., Shao X., Zhu L., Huang K., Luo Y. (2017). A rapid and visual aptasensor for lipopolysaccharides detection based on the bulb-like triplex turn-on switch coupled with HCR-HRP nanostructures. Biosens. Bioelectron..

[109] Bai L., Chai Y., Pu X., Yuan R. (2014). A signal-on electrochemical aptasensor for ultrasensitive detection of endotoxin using three-way DNA junction-aided enzymatic recycling and graphene nanohybrid for amplification. Nanoscale.

[110] Chen C., Xie Q., Yang D., Xiao H., Fu Y., Tan Y., Yao S. (2013). Recent advances in electrochemical glucose biosensors: A review. RSC Adv..

[111] Miao P. (2013). Electrochemical sensing strategies for the detection of endotoxin: A review. RSC Adv..

[112] Wright S.D., Ramos R.A., Tobias P.S., Ulevitch R.J., Mathison J.C. (1990). CD14, a receptor for complexes of lipopolysaccharide (LPS) and LPS binding protein. Science.

[113] Opal S.M., Scannon P.J., Vincent J.L., White M., Carroll S.F., Palardy J.E., Parejo N.A., Pribble J.P., Lemke J.H. (1999). Relationship between plasma levels of lipopolysaccharide (LPS) and LPS-binding protein in patients with severe sepsis and septic shock. J. Infect. Dis..

[114] Lan M., Wu J., Liu W., Zhang W., Ge J., Zhang H., Sun J., Zhao W., Wang P. (2012). Copolythiophene-derived colorimetric and fluorometric sensor for visually supersensitive determination of lipopolysaccharide. J. Am. Chem. Soc..

[115] Liu L., Jin H., Sun L., Ma S., Lin R. (2012). Determination of Aflatoxins in Medicinal Herbs by High-performance Liquid Chromatography–Tandem Mass Spectrometry. Phytochem. Analusis.

[116] Kolosova A.Y., Shim W.B., Yang Z.Y., Eremin S.A., Chung D.H. (2006). Direct competitive ELISA based on a monoclonal antibody for detection of aflatoxin B 1. Stabilization of ELISA kit components and application to grain samples. Anal. Bioanal. Chem..

[117] Chen F., Luan C., Wang L., Wang S., Shao L. (2017). Simultaneous determination of six mycotoxins in peanut by high-performance liquid chromatography with a fluorescence detector. J. Sci. Food Agric..

[118] Geleta G.S., Zhao Z., Wang Z. (2018). A novel reduced graphene oxide/molybdenum disulfide/polyaniline nanocomposite-based electrochemical aptasensor for detection of aflatoxin B_1_. Analyst.

[119] Roushani M., Nezhadali A., Jalilian Z. (2018). An electrochemical chlorpyrifos aptasensor based on the use of a glassy carbon electrode modified with an electropolymerized aptamer-imprinted polymer and gold nanorods. Microchim. Acta.

[120] Liu S., Wang Y., Xu W., Leng X., Wang H., Guo Y., Huang J. (2017). A novel sandwich-type electrochemical aptasensor based on GR-3D Au and aptamer-AuNPs-HRP for sensitive detection of oxytetracycline. Biosens. Bioelectron..

[121] Shrivas K., Shankar R., Dewangan K. (2015). Gold nanoparticles as a localized surface plasmon resonance based chemical sensor for on-site colorimetric detection of arsenic in water samples. Sens. Actuators B Chem..

[122] Cui H., Yang W., Li X., Zhao H., Yuan Z. (2012). An electrochemical sensor based on a magnetic Fe_3_O_4_ nanoparticles and gold nanoparticles modified electrode for sensitive determination of trace amounts of arsenic (III). Anal. Methods.

[123] Ensafi A.A., Kazemifard N., Rezaei B. (2016). A simple and sensitive fluorimetricaptasensor for the ultrasensitive detection of arsenic (III) based on cysteamine stabilized CdTe/ZnS quantum dots aggregation. Biosens. Bioelectron..

[124] Song L., Mao K., Zhou X., Hu J. (2016). A novel biosensor based on Au@ Ag core–shell nanoparticles for SERS detection of arsenic (III). Talanta.

[125] Pooja D., Saini S., Thakur A., Kumar B., Tyagi S., Nayak M.K. (2017). A “Turn-On” thiol functionalized fluorescent carbon quantum dot based chemosensory system for arsenite detection. J. Hazard. Mater..

[126] Vadahanambi S., Lee S.H., Kim W.J., Oh I.K. (2013). Arsenic removal from contaminated water using three-dimensional graphene-carbon nanotube-iron oxide nanostructures. Environ. Sci. Technol..

[127] Wen S., Zhang C., Liang R., Chi B., Yuan Y., Qiu J. (2017). Highly sensitive voltammetric determination of arsenite by exploiting arsenite-induced conformational change of ssDNA and the electrochemical indicator Methylene Blue. Microchim. Acta.

[128] Gao Y., Cao X., Jingjing Y.U., Wang X. (2009). Determination of arsenic and its species in dry seafood by high performance liquid chromatography-inductively coupled plasma mass spectrometry. Chin. J. Anal. Chem..

[129] Zhang N., Fu N., Fang Z., Feng Y., Ke L. (2011). Simultaneous multi-channel hydride generation atomic fluorescence spectrometry determination of arsenic, bismuth, tellurium and selenium in tea leaves. Food Chem..

[130] Hassanpoor S., Khayatian G., Azar A.R.J. (2015). Ultra-trace determination of arsenic species in environmental waters, food and biological samples using a modified aluminum oxide nanoparticle sorbent and AAS detection after multivariate optimization. Microchim. Acta.

[131] Rabieh S., Bagheri M., Planer-Friedrich B. (2013). Speciation of arsenite and arsenate by electrothermal AAS following ionic liquid dispersive liquid-liquid microextraction. Microchim. Acta.

[132] Wang H., Yuan X., Zeng G., Wu Y., Liu Y., Jiang Q., Gu S. (2015). Three dimensional graphene based materials: Synthesis and applications from energy storage and conversion to electrochemical sensor and environmental remediation. Adv. Colloid Interface Sci..

[133] Zhang Z., Ji H., Song Y., Zhang S., Wang M., Jia C., Tian J.Y., He L., Zhang X., Liu C.S. (2017). Fe (III)-based metal–organic framework-derived core–shell nanostructure: Sensitive electrochemical platform for high trace determination of heavy metal ions. Biosens. Bioelectron..

[134] Ensafi A.A., Akbarian F., Heydari-Soureshjani E., Rezaei B. (2018). A novel aptasensor based on 3D-reduced graphene oxide modified gold nanoparticles for determination of arsenite. Biosens. Bioelectron..

[135] Fritzen-Garcia M.B., Monteiro F.F., Cristofolini T., Acuña J.J.S., Zanetti-Ramos B.G., Oliveira I.R.W., Soldi V., Pasa A.A., Creczynski-Pasa T.B. (2013). Characterization of horseradish peroxidase immobilized on PEGylated polyurethane nanoparticles and its application for dopamine detection. Sens. Actuators B Chem..

[136] Qian T., Yu C., Zhou X., Ma P., Wu S., Xu L., Shen J. (2014). Ultrasensitive dopamine sensor based on novel molecularly imprinted polypyrrole coated carbon nanotubes. Biosens. Bioelectron..

[137] Venton B.J., Wightman R.M. (2003). Psychoanalytical electrochemistry: Dopamine and behavior. Anal. Chem..

[138] Shahzad F., Zaidi S.A., Koo C.M. (2017). Highly sensitive electrochemical sensor based on environmentally friendly biomass-derived sulfur-doped graphene for cancer biomarker detection. Sens. Actuators B Chem..

[139] Sheng Z.H., Zheng X.Q., Xu J.Y., Bao W.J., Wang F.B., Xia X.H. (2012). Electrochemical sensor based on nitrogen doped graphene: Simultaneous determination of ascorbic acid, dopamine and uric acid. Biosens. Bioelectron..

[140] Sassolas A., Blum L.J., Leca-Bouvier B.D. (2009). Electrochemical aptasensors. Electroanal. Int. J. Devoted Fundam. Pract. Asp. Electroanal..

[141] Deng C.Y., Chen J.H., Nie Z., Wang M.D., Chu X.C., Chen X.L., Xiao X.L., Lei C.Y., Yao S.Z. (2009). Impedimetric aptasensor with femtomolar sensitivity based on the enlargement of surface-charged gold nanoparticles. Anal. Chem..

[142] Chen R.J., Zhang Y.G., Wang D.W., Dai H.J. (2001). Noncovalent sidewall functionalization of single-walled carbon 381 nanotubes for protein immobilization. J. Am. Chem. Soc..

[143] Zhao C.Q., Jin H., Gui R.J., Wang Z.H. (2017). Facile fabrication of dual-ratiometric electrochemical sensors based on a bare electrode for dual-signal sensing of analytes in electrolyte solution. Sens. Actuators B Chem..

[144] Hou L., Zhang X., Kong M., Jiang G., Sun Y., Mo W., Lin T., Ye F., Zhao S. (2020). A competitive immunoassay for electrochemical impedimetric determination of chlorpyrifos using a nanogold-modified glassy carbon electrode based on enzymatic biocatalytic precipitation. Microchim. Acta.

[145] Jin H., Zhao C., Gui R., Gao X., Wang Z. (2018). Reduced graphene oxide/nile blue/gold nanoparticles complex-modified glassy carbon electrode used as a sensitive and label-free aptasensor for ratiometric electrochemical sensing of dopamine. Anal. Chim. Acta.

[146] Chen W., Liu Y., Zhang Y., Fang J., Xu P., Xu J., Li X., Liu C.C., Wen W. (2017). Highly effective and specific way for the trace analysis of carbaryl insecticides based on Au 42 Rh 58 alloy nanocrystals. J. Mater. Chem. A.

[147] Ruan C., Yang L., Li Y. (2002). Rapid detection of viable Salmonella typhimurium in a selective medium by monitoring oxygen consumption with electrochemical cyclic voltammetry. J. Electroanal. Chem..

[148] Berg J.D., Fiksdal L. (1988). Rapid detection of total and fecal coliforms in water by enzymatic hydrolysis of 4-methylumbelliferone-beta-D-galactoside. Appl. Environ. Microbiol..

[149] Tryland I., Fiksdal L. (1998). Enzyme characteristics of β-d-galactosidase-and β-d-glucuronidase-positive bacteria and their interference in rapid methods for detection of waterborne coliforms and Escherichia coli. Appl. Environ. Microbiol..

[150] Bhatnagar-Mathur P., Sunkara S., Bhatnagar-Panwar M., Waliyar F., Sharma K.K. (2015). Biotechnological advances for combating Aspergillus flavus and aflatoxin contamination in crops. Plant Sci..

[151] Bhatnagar D., Rajasekaran K., Payne G., Brown R., Yu J., Cleveland T. (2008). The ‘omics’ tools: Genomics, proteomics, metabolomics and their potential for solving the aflatoxin contamination problem. World Mycotoxin J..

[152] Bergholz T.M., Switt A.I.M., Wiedmann M. (2014). Omics approaches in food safety: Fulfilling the promise?. Trends Microbiol..

[153] Garcia-Cela E., Verheecke-Vaessen C., Magan N., Medina A. (2018). The “-omics” contributions to the understanding of mycotoxin production under diverse environmental conditions. Curr. Opin. Food Sci..

[154] Leng Y., Sun K., Chen X., Li W. (2015). Suspension arrays based on nanoparticle-encoded microspheres for high-throughput multiplexed detection. Chem. Soc. Rev..

[155] Anfossi L., Giovannoli C., Baggiani C. (2016). Mycotoxin detection. Curr. Opin. Biotechnol..

[156] Agriopoulou S., Stamatelopoulou E., Varzakas T. (2020). Advances in analysis and detection of major mycotoxins in foods. Foods.

[157] Righetti L., Dall’Asta C., Hajslova J., Rubert J. (2016). Metabolomics approaches and their hidden potential for explaining the mycotoxin contamination problem. Metabolomics:Fundamentals and Applications.

[158] Balmer D., Flors V., Glauser G., Mauch-Mani B. (2013). Metabolomics of cereals under biotic stress: Current knowledge and techniques. Front. Plant Sci..

[159] Gauthier L., Atanasova-Penichon V., Chéreau S., Richard-Forget F. (2015). Metabolomics to decipher the chemical defense of cereals against Fusarium graminearum and deoxynivalenol accumulation. Int. J. Mol. Sci..

[160] Varga E., Glauner T., Berthiller F., Krska R., Schuhmacher R., Sulyok M. (2013). Development and validation of a (semi-) quantitative UHPLC-MS/MS method for the determination of 191 mycotoxins and other fungal metabolites in almonds, hazelnuts, peanuts and pistachios. Anal. Bioanal. Chem..

[161] Mahmoud M.A. (2015). Detection of Aspergillus flavus in Stored Peanuts Using Real-Time PCR and the Expression of Aflatoxin Genes in Toxigenic and Atoxigenic A. flavus Isolates. Foodborne Pathog. Dis..

[162] Zhang J., Chiodini R., Badr A., Zhang G. (2011). The impact of next-generation sequencing on genomics. J. Genet. Genom..

[163] Yu J., Ronning C.M., Wilkinson J.R., Campbell B.C., Payne G.A., Bhatnagar D., Cleveland T.E., Nierman W.C. (2007). Gene profiling for studying the mechanism of aflatoxin biosynthesis in *Aspergillus flavus* and *A. parasiticus*. Food Addit. Contam..

[164] Moore G.G., Mack B.M., Beltz S.B. (2015). Genomic sequence of the aflatoxigenic filamentous fungus Aspergillus nomius. BMC Genom..

[165] Kim J.H., Yu J., Mahoney N., Chan K.L., Molyneux R.J., Varga J., Bhatnagar D., Cleveland T.E., Nierman W.C., Campbell B.C. (2008). Elucidation of the functional genomics of antioxidant-based inhibition of aflatoxin biosynthesis. Int. J. Food Microbiol..

[166] Cleveland T.E., Bhatnagar D., Yu J., Appell M., Kendra F.D.F., Trucksess M.W. (2010). Elimination and control of aflatoxin contamination in agricultural crops through Aspergillus flavus genomics. Mycotoxin Prevention and Control in Agriculture.

[167] Faustinelli P.C., Wang X.M., Palencia E.R., Arias R.S. (2016). Genome sequences of eight *Aspergillus flavus* spp. and one *A. parasiticus* sp., isolated from peanut seeds in georgia. Genome Announc..

[168] Pedrotty D.M., Morley M.P., Cappola T.P. (2012). Transcriptomic biomarkers of cardiovascular disease. Prog. Cardiovasc. Dis..

[169] Macaulay I.C., Carr P., Gusnanto A., Ouwehand W., Fitzgerald D., Watkins N. (2005). Platelet genomics and proteomics in human health and disease. J. Clin. Investig..

[170] Rychlik M., Kanawati B., Schmitt-Kopplin P. (2017). Foodomics as a promising tool to investigate the mycobolome. TrAC Trends Anal. Chem..

[171] Alwine J.C., Kemp D.J., Stark G.R. (1977). Method for detection of specific RNAs in agarose gels by transfer to diazobenzyloxymethyl-paper and hybridization with DNA probes. Proc. Natl. Acad. Sci. USA.

[172] Malone J.H., Oliver B. (2011). Microarrays, deep sequencing and the true measure of the transcriptome. BMC Biol..

[173] Lowe R., Shirley N., Bleackley M., Dolan S., Shafee T. (2017). Transcriptomics technologies. PLoS Comput. Biol..

[174] Castellá G., Bragulat M.R., Puig L., Sanseverino W., Cabañes F.J. (2018). Genomic diversity in ochratoxigenic and non ochratoxigenic strains of *Aspergillus carbonarius*. Sci. Rep..

[175] Musungu B.M., Bhatnagar D., Brown R.L., Payne G.A., OBrian G., Fakhoury A.M., Geisler M. (2016). A Network Approach of Gene Co-Expression in the Zea mays/Aspergillus flavus Pathosystem to Map Host/Pathogen Interaction Pathways. Front. Genet..

[176] Nayak S.N., Agarwal G., Pandey M.K., Sudini H.K., Jayale A.S., Purohit S., Desai A., Wan L., Guo B., Liao B. (2017). Aspergillus flavus infection triggered immune responses and host-pathogen cross-talks in groundnut during in-vitro seed colonization. Sci. Rep..

[177] Bhatnagar D., Rajasekaran K., Gilbert M., Cary J.W., Magan N. (2018). Advances in molecular and genomic research to safeguard food and feed supply from aflatoxin contamination. World Mycotoxin J..

[178] Vettorazzi A., van Delft J., López de Cerain A. (2013). A review on ochratoxin: A transcriptomic studies. Food Chem. Toxicol..

[179] Eshelli M., Qader M.M., Jambi E.J., Hursthouse A.S., Rateb M.E. (2018). Current status and future opportunities of omics tools in mycotoxin research. Toxins.

[180] Levin R.E. (2012). PCR detection of aflatoxin producing fungi and its limitations. Int. J. Food Microbiol..

[181] Notomi T., Okayama H., Masubuchi H., Yonekawa T., Watanabe K., Amino N., Hase T. (2018). Loop-mediated isothermal amplification of DNA. Nucleic Acids Res..

[182] Kaneko H., Kawana T., Fukushima E., Suzutani T. (2007). Tolerance of loop-mediated isothermal amplification to a culture medium and biological substances. J. Biochem. Biophys. Methods.

[183] Nagamine K., Hase T., Notomi T. (2002). Accelerated reaction by loop-mediated isothermal amplification using loop primers. Mol. Cell. Probes.

[184] Niessen L., Bechtner J., Fodil S., Taniwaki M.H., Vogel R.F. (2018). LAMP-based group specific detection of aflatoxin producers within Aspergillus section Flavi in food raw materials, spices, and dried fruit using neutral red for visible-light signal detection. Int. J. Food Microbiol..

[185] Niessen L., Luo J., Denschlag C., Vogel R.F. (2013). The application of loop-mediated isothermal amplification (LAMP) in food testing for bacterial pathogens and fungal contaminants. Food Microbiol..

[186] Parida M., Sannarangaiah S., Dash P.K., Rao P., Morita K. (2008). Loop mediated isothermal amplification (LAMP): A new generation of innovative gene amplification techniques; perspectives in clinical diagnosis of infectious diseases. Rev. Med. Virol..

[187] Niessen L. (2015). Current state and future perspectives of loop-mediated isothermal amplification (LAMP)-based diagnosis of filamentous fungi and yeasts. Appl. Microbiol. Biotechnol..

[188] Luo J., Taniwaki M.H., Iamanaka B.T., Vogel R.F., Niessen L. (2014). Application of loop-mediated isothermal amplification assays for direct identification of pure cultures of *Aspergillus flavus*, *A. nomius*, and *A. caelatus* and for their rapid detection in shelled Brazil nuts. Int. J. Food Microbiol..

[189] Luo J., Vogel R.F., Niessen L. (2012). Development and application of a loop-mediated isothermal amplification assay for rapid identification of aflatoxigenic molds and their detection in food samples. Int. J. Food Microbiol..

[190] Luo J., Vogel R.F., Niessen L. (2014). Rapid detection of aflatoxin producing fungi in food by real-time quantitative loop-mediated isothermal amplification. Food Microbiol..

[191] Reddy K., Reddy C., Muralidharan K. (2009). Detection of Aspergillus spp. and aflatoxin B_1_ in rice in India. Food Microbiol..

[192] Romero M.R., Cook N.A. (2018). Rapid LAMP-Based Method for Screening Poultry Samples for Campylobacter without Enrichment. Front Microbiol..

[193] Wang P. (2021). Nucleic acid-based rapid methods for the detection of foodborne pathogens. Journal of Physics: Conference Series.

[194] Sadhasivam S., Britzi M., Zakin V., Kostyukovsky M., Trostanetsky A., Quinn E., Sionov E. (2017). Rapid detection and identification of mycotoxigenic fungi and mycotoxins in stored wheat grain. Toxins.

[195] Dong T., Sun J., Liu B., Zhang Y., Song Y., Wang S. (2010). Development of a sensitivity-improved immunoassay for the determination of carbaryl in food samples. J. Sci. Food Agric..

[196] Wang S., Yu C.D., Wang J.P. (2005). Enzyme immunoassay for the determination of carbaryl residues in agricultural products. Food Addit. Contam..

[197] Zhang C., Ma G., Fang G., Zhang Y., Wang S. (2008). Development of a capillary electrophoresis-based immunoassay with laser-induced fluorescence for the detection of carbaryl in rice samples. J. Agric. Food Chem..

[198] Nasir M.Z.M., Mayorga-Martinez C.C., Sofer Z., Pumera M. (2017). Two-dimensional 1T-phase transition metal dichalcogenides as nanocarriers to enhance and stabilize enzyme activity for electrochemical pesticide detection. ACS Nano.

[199] Croci L., Delibato E., Volpe G., De Medici D., Palleschi G. (2004). Comparison of PCR, electrochemical enzyme-linked immunosorbent assays, and the standard culture method for detecting Salmonella in meat products. Appl. Environ. Microbiol..

[200] Brewster J.D., Mazenko R.S. (1998). Filtration capture and immunoelectrochemical detection for rapid assay of Escherichia coli O157: H7. J. Immunol. Methods.

[201] Lazcka O., Del Campo F.J., Munoz F.X. (2007). Pathogen detection: A perspective of traditional methods and biosensors. Biosens. Bioelectron..

[202] Mittelmann A.S., Ron E.Z., Rishpon J. (2002). Amperometric Quantification of Total Coliforms and Specific Detection of Escherichia coli. Anal. Chem..

[203] Bodur S., Özlü C., Tışlı B., Fırat M., Chormey D.S., Bakırdere S. (2020). Analytical protocol for determination of endosulfan beta, propham, chlorpyrifos, and acibenzolar-s-methyl in lake water and wastewater samples by gas chromatography–mass spectrometry after dispersive liquid–liquid microextraction. Environ. Monit. Assess..

[204] Crutchfield C.A., Lu W., Melamud E., Rabinowitz J.D. (2010). Mass spectrometry-based metabolomics of yeast. Methods Enzymol..

[205] Santos I.C., Schug K.A. (2017). Recent advances and applications of gas chromatography vacuum ultraviolet spectroscopy. J. Sep. Sci..

[206] Stashenko E., Ren J. (2014). Advances in Gas Chromatography.

[207] Viñas P., Campillo N., Andruch V. (2015). Recent achievements in solidified floating organic drop microextraction. TrAC Trends Anal. Chem..

[208] Zeng C., Ji L., Zhou C., Zhang F., Liu M., Xie Q. (2015). Chemical vapor generation of bismuth in non-aqueous phase based on cloud point extraction coupled with thermospray flame furnace atomic absorption spectrometric determination. Microchem. J..

[209] Román I.P., Chisvert Alberto A., Canals A. (2011). Dispersive solid-phase extraction basedo n oleic acid-coated magnetic nanoparticles followed by gas chromatography mass spectrometry for UV-filter determination in water samples. J. Chromatogr. A.

[210] Xiong J., Hu B. (2008). Comparison of hollow fiber liquid phase microextraction and dispersive liquid-liquid microextraction for the determination of organosulfur pesticides in environmental and beverage samples by gas chromatography with flame photometric detection. J. Chromatogr. A.

[211] Šrámková I.H., Horstkotte B., Fikarová K., Sklenářová H., Solich P. (2018). Direct-immersion single-drop microextraction and in-drop stirring microextraction for the determination of nanomolar concentrations of lead using automated Lab-In-Syringe technique. Talanta.

[212] Vidal M., Ospina M., Serafim A.B., Calafat A.M., Baker S.E., Morales-Agudelo P. (2018). Quantification of DEET and neonicotinoid pesticide biomarkers in human urine by online solid-phase extraction high-performance liquid chromatography-tandem mass spectrometry. Anal. Bioanal. Chem..

[213] Rezaee M., Assadi Y., Milani Hosseini M.R., Aghaee E., Ahmadi F., Berijani S. (2006). Determination of organic compounds in water using dispersive liquid-liquid microextraction. J. Chromatogr. A.

[214] Rykowska I., Ziemblińska J., Nowak I. (2018). Modern approaches in dispersive liquid-liquid microextraction (DLLME) based on ionic liquids: A review. J. Mol. Liq..

[215] Armenta S., Garrigues S., de la Guardia M. (2008). Green analytical chemistry. TrAC Trends Anal. Chem..

[216] Ranjbari E., Hadjmohammadi M.R., Kiekens F., De Wael K. (2015). Mixed Hemi/Ad-Micelle Sodium Dodecyl Sulfate-Coated Magnetic Iron Oxide Nanoparticles for the Efficient Removal and Trace Determination of Rhodamine-B and Rhodamine-6G. Anal. Chem..

[217] Castilho M.D.S., Laube T., Yamanaka H., Alegret S., Pividori M.I. (2011). Magneto Immunoassays for Plasmodium falciparum Histidine-Rich. Anal. Chem..

[218] Chaichi M.J., Ehsani M. (2016). A novel glucose sensor based on immobilization of glucose oxidase on the chitosan-coated Fe_3_O_4_ nanoparticles and the luminol-H_2_O_2_ -gold nanoparticle chemiluminescence detection system. Sens. Actuators B Chem..

[219] Medina-Sánchez M., Mayorga-Martinez C.C., Watanabe T., Ivandini T.A., Honda Y., Pino F., Nakata A., Fujishima A., Einaga Y., Merkoçi A. (2016). Microfluidic platform for environmental contaminants sensing and degradation based on boron-doped diamond electrodes. Biosens. Bioelectron..

[220] Rodrigues N.F.M., Neto S.Y., Luz R.D.C.S., Damos F.S., Yamanaka H. (2018). Ultrasensitive determination of malathion using acetylcholinesterase immobilized on chitosan-functionalized magnetic iron nanoparticles. Biosensors.

[221] Bunney J., Williamson S., Atkin D., Jeanneret M., Cozzolino D., Chapman J., Power A., Chandra S. (2017). The use of electrochemical biosensors in food analysis. Curr. Res. Nutr. Food Sci. J..

[222] Lucarelli F., Marrazza G., Turner A.P.F., Mascini M. (2004). Carbon and gold electrodes as electrochemical transducers for DNA hybridisation sensors. Biosens. Bioelectron..

[223] Kerman K., Vestergaard M.D., Nagatani N., Takamura Y., Tamiya E. (2006). Electrochemical genosensor based on peptide nucleic acid-mediated PCR and asymmetric PCR techniques: Electrostatic interactions with a metal cation. Anal. Chem..

[224] Palecek E., Fojta M. (2005). Electrochemical DNA sensors. Bioelectronics: From Theory to Applications.

[225] Wang J. (2002). Electrochemical nucleic acid biosensors. Anal. Chim. Acta.

[226] Pividori M.I., Merkoci A., Alegret S. (2002). Electrochemical genosensor design: Immobilisation of oligonucleotides onto transducer surfaces and detection methods. Biosens. Bioelectron..

[227] Farabullini F., Lucarelli F., Palchetti I., Marrazza G., Mascini M. (2007). Disposable electrochemical genosensor for the simultaneous analysis of different bacterial food contaminants. Biosens. Bioelectron..

[228] Lermo A., Campoy S., Barbe J., Hernandez S., Alegret S., Pividori M.I. (2007). In situ DNA amplification with magnetic primers for the electrochemical detection of food pathogens. Biosens. Bioelectron..

[229] Elsholz B., Wörl R., Blohm L., Albers J., Feucht H., Grunwald T., Jurgen T., Schweder T., Hintsche R. (2006). Automated detection and quantitation of bacterial RNA by using electrical microarrays. Anal. Chem..

[230] Baeumner A.J., Cohen R.N., Miksic V., Min J. (2003). RNA biosensor for the rapid detection of viable Escherichia coli in drinking water. Biosens. Bioelectron..

[231] Alsammarraie F.K., Lin M. (2017). Using standing gold nanorod arrays as surface-enhanced Raman spectroscopy (SERS) substrates for detection of carbaryl residues in fruit juice and milk. J. Agric. Food Chem..

[232] Ma Z., Chen P., Cheng W., Yan K., Pan L., Shi Y., Yu G. (2018). Highly sensitive, printable nanostructured conductive polymer wireless sensor for food spoilage detection. Nano Lett..

[233] Rateni G., Dario P., Cavallo F. (2017). Smartphone-based food diagnostic technologies: A review. Sensors.

[234] Zhang D., Liu Q. (2016). Bioelectronics: Biosensors and bioelectronics on smartphone for portable biochemical detection. Biosens. Bioelectron..

[235] Ross G.M.S., Bremer M.G.E.G., Nielen M. (2018). Consumer-friendly food allergen detection: Moving towards smartphone-based immunoassays. Anal. Bioanal. Chem..

[236] Lillehoj P.B., Huang M.C., Truong N., Ho C.M. (2013). Rapid electrochemical detection on a mobile phone. Lab Chip.

[237] Rotariu L., Lagarde F., Jaffrezic-Renault N., Bala C. (2016). Electrochemical biosensors for fast detection of food contaminants–trends and perspective. TrAC Trends Anal. Chem..

[238] Li L., Pan L., Ma Z., Yan K., Cheng W., Shi Y., Yu G. (2018). All inkjet-printed amperometric multiplexed biosensors based on nanostructured conductive hydrogel electrodes. Nano Lett..

[239] Quesada-González D., Merkoçi A. (2017). Bioelectronics: Mobile phone-based biosensing: An emerging “diagnostic and communication” technology. Biosens. Bioelectron..

[240] Liu J., Geng Z., Fan Z., Liu J., Chen H. (2019). Point-of-care testing based on smartphone: The current state-of-the-art (2017–2018). Biosens. Bioelectron..

[241] Li S., Liu J., Chen Z., Lu Y., Low S.S., Zhu L., Cheng C., He Y., Chen Q., Su B. (2019). Electrogenerated chemiluminescence on smartphone with graphene quantum dots nanocomposites for Escherichia Coli detection. Sens. Actuators B Chem..

[242] Cheng N., Song Y., Zeinhom M.M., Chang Y.C., Sheng L., Li H., Du D., Li L., Zhu M.J., Luo Y. (2017). Nanozyme-mediated dual immunoassay integrated with smartphone for use in simultaneous detection of pathogens. ACS Appl. Mater. Interfaces.

[243] Shan Y., Wang B., Huang H., Jian D., Wu X., Xue L., Wang S., Liu F. (2019). On-site quantitative Hg2+ measurements based on selective and sensitive fluorescence biosensor and miniaturized smartphone fluorescence microscope. Biosens. Bioelectron..

[244] Chen X., Mei Q., Yu L., Ge H., Yue J., Zhang K., Hayat T., Alsaedi A., Wang S. (2018). Rapid and onsite detection of uranyl ions via ratiometric fluorescence signals based on a smartphone platform. ACS Appl. Mater. Interfaces.

[245] GSMA The Mobile Economy 2019. https://www.gsma.com/mobileeconomy/wp-content/uploads/2021/07/GSMA_MobileEconomy2021_3.pdf.

[246] Zeinhom M.M.A., Wang Y., Sheng L., Du D., Li L., Zhu M.J., Lin Y. (2018). Smart phone based immunosensor coupled with nanoflower signal amplification for rapid detection of Salmonella Enteritidis in milk, cheese and water. Sens. Actuators B Chem..

[247] Zhang J., Khan I., Zhang Q., Liu X., Dostalek J., Liedberg B., Wang Y. (2018). Lipopolysaccharides detection on a grating-coupled surface plasmon resonance smartphone biosensor. Biosens. Bioelectron..

[248] Zheng L., Cai G., Wang S., Liao M., Li Y., Lin J. (2019). Bioelectronics: A microfluidic colorimetric biosensor for rapid detection of Escherichia coli O157: H7 using gold nanoparticle aggregation and smart phone imaging. Biosens. Bioelectron..

[249] Zhang H., Xue L., Huang F., Wang S., Wang L., Liu N., Lin J. (2019). A capillary biosensor for rapid detection of Salmonella using Fe-nanocluster amplification and smart phone imaging. Biosens. Bioelectron..

[250] Wang S., Zheng L., Cai G., Liu N., Liao M., Li Y., Zhang X., Lin J. (2019). A microfluidic biosensor for online and sensitive detection of Salmonella typhimurium using fluorescence labeling and smartphone video processing. Biosens. Bioelectron..

[251] Li Z., Zhang S., Yu T., Dai Z., Wei Q. (2019). Aptamer-based fluorescent sensor array for multiplexed detection of cyanotoxins on a smartphone. Anal. Chem..

[252] Hu X., Shi J., Shi Y., Zou X., Arslan M., Zhang W., Huang X., Li Z., Xu Y. (2019). Use of a smartphone for visual detection of melamine in milk based on Au@ carbon quantum dots nanocomposites. Food Chem..

[253] Xu G., Cheng C., Liu Z., Yuan W., Wu X., Lu Y., Low S.S., Liu J., Zhu L., Ji D. (2019). Battery-Free and Wireless Epidermal Electrochemical System with All-Printed Stretchable Electrode Array for Multiplexed In Situ Sweat Analysis. Adv. Mater. Technol..

[254] Ji D., Liu L., Li S., Chen C., Lu Y., Wu J., Liu Q. (2017). Bioelectronics: Smartphone-based cyclic voltammetry system with graphene modified screen printed electrodes for glucose detection. Biosens. Bioelectron..

[255] Xu G., Cheng C., Yuan W., Liu Z., Zhu L., Li X., Lu Y., Chen Z., Liu J., Cui Z. (2019). Smartphone-based battery-free and flexible electrochemical patch for calcium and chloride ions detections in biofluids. Sens. Actuators B Chem..

[256] Zhang D., Jiang J., Chen J., Zhang Q., Lu Y., Yao Y., Li S., Liu G.L., Liu Q. (2015). Smartphone-based portable biosensing system using impedance measurement with printed electrodes for 2, 4, 6-trinitrotoluene (TNT) detection. Biosens. Bioelectron..

[257] Lin H.Y., Huang C.H., Park J., Pathania D., Castro C.M., Fasano A., Weissleder R., Lee H. (2017). Integrated magneto-chemical sensor for on-site food allergen detection. ACS Nano.

[258] Li S., Zhang D., Liu J., Cheng C., Zhu L., Li C., Lu Y., Low S.S., Su B., Liu Q. (2019). Electrochemiluminescence on smartphone with silica nanopores membrane modified electrodes for nitroaromatic explosives detection. Biosens. Bioelectron..

[259] Cheng N., Song Y., Fu Q., Du D., Luo Y., Wang Y., Xu W., Lin Y. (2018). Aptasensor based on fluorophore-quencher nano-pair and smartphone spectrum reader for on-site quantification of multi-pesticides. Biosens. Bioelectron..

[260] Mishra R.K., Hubble L.J., Martín A., Kumar R., Barfidokht A., Kim J., Musameh M.M., Kyratzis I.L., Wang J. (2017). Wearable flexible and stretchable glove biosensor for on-site detection of organophosphorus chemical threats. ACS Sens..

[261] Lu Y., Shi Z., Liu Q. (2019). Smartphone-based biosensors for portable food evaluation. Curr. Opin. Food Sci..

[262] Zeinhom M.M.A., Wang Y., Song Y., Zhu M.J., Lin Y., Du D. (2018). A portable smart-phone device for rapid and sensitive detection of E. coli O157:H7 in Yoghurt and Egg. Biosens. Bioelectron..

[263] Wilson W.J., Strout C.L., DeSantis T.Z., Stilwell J.L., Carrano A.V., Andersen G.L. (2002). Sequence-specific identification of 18 pathogenic microorganisms using microarray technology. Mol. Cell. Probes.

[264] Mockler T.C., Ecker J.R. (2005). Applications of DNA tiling arrays for whole-genome analysis. Genomics.

[265] Bertone P., Gerstein M., Snyder M. (2005). Applications of DNA tiling arrays to experimental genome annotation and regulatory pathway discovery. Chromosome Res..

[266] Hu W., Feng K., Jiang A., Xiu Z., Lao Y., Li Y., Long Y. (2020). An In Situ-Synthesized Gene Chip for the Detection of Food-Borne Pathogens on Fresh-Cut Cantaloupe and Lettuce. Front. Microbiol..

[267] Shridhar P.B., Patel I.R., Gangiredla J., Noll L.W., Shi X., Bai J., Elkins C.A., Strockbine N., Nagaraja T.G. (2018). DNA microarray-based assessment of virulence potential of Shiga toxin gene-carrying Escherichia coli O104: H7 isolated from feedlot cattle feces. PLoS ONE.

[268] Figueiredo R., Card R., Nunes C., AbuOun M., Bagnall M.C., Nunez J., Mendonca N., Anjum M.F., Silva G.J.D. (2015). Virulence Characterization of Salmonella enterica by a New Microarray: Detection and Evaluation of the Cytolethal Distending Toxin Gene Activity in the Unusual Host S. Typhimurium. PLoS ONE.

